# Year-Round Monitoring of Contaminants in Neal and Rogers Creeks, Hood River Basin, Oregon, 2011-12, and Assessment of Risks to Salmonids

**DOI:** 10.1371/journal.pone.0158175

**Published:** 2016-06-27

**Authors:** Whitney B. Hapke, Jennifer L. Morace, Elena B. Nilsen, David A. Alvarez, Kevin Masterson

**Affiliations:** 1 U.S. Geological Survey, Oregon Water Science Center, Portland, Oregon, United States of America; 2 U.S. Geological Survey, Columbia Environmental Research Center, Columbia, Missouri, United States of America; 3 Oregon Department of Environmental Quality, Bend, Oregon, United States of America; Institute for Health & the Environment, UNITED STATES

## Abstract

Pesticide presence in streams is a potential threat to Endangered Species Act listed salmonids in the Hood River basin, Oregon, a primarily forested and agricultural basin. Two types of passive samplers, polar organic chemical integrative samplers (POCIS) and semipermeable membrane devices (SPMDs), were simultaneously deployed at four sites in the basin during Mar. 2011–Mar. 2012 to measure the presence of pesticides, polybrominated diphenyl ethers (PBDEs), and polychlorinated biphenyls (PCBs). The year-round use of passive samplers is a novel approach and offers several new insights. Currently used pesticides and legacy contaminants, including many chlorinated pesticides and PBDEs, were present throughout the year in the basin’s streams. PCBs were not detected. Time-weighted average water concentrations for the 2-month deployment periods were estimated from concentrations of chemicals measured in the passive samplers. Currently used pesticide concentrations peaked during spring and were detected beyond their seasons of expected use. Summed concentrations of legacy contaminants in Neal Creek were highest during July–Sept., the period with the lowest streamflows. Endosulfan was the only pesticide detected in passive samplers at concentrations exceeding Oregon or U.S. Environmental Protection Agency water-quality thresholds. A Sensitive Pesticide Toxicity Index (SPTI) was used to estimate the relative acute potential toxicity among sample mixtures. The acute potential toxicity of the detected mixtures was likely greater for invertebrates than for fish and for all samples in Neal Creek compared to Rogers Creek, but the indices appear to be low overall (<0.1). Endosulfans and pyrethroid insecticides were the largest contributors to the SPTIs for both sites. SPTIs of some discrete (grab) samples from the basin that were used for comparison exceeded 0.1 when some insecticides (azinphos methyl, chlorpyrifos, malathion) were detected at concentrations near or exceeding acute water-quality thresholds. Early life stages and adults of several sensitive fish species, including salmonids, are present in surface waters of the basin throughout the year, including during periods of peak estimated potential toxicity. Based on these data, direct toxicity to salmonids from in-stream pesticide exposure is unlikely, but indirect impacts (reduced fitness due to cumulative exposures or negative impacts to invertebrate prey populations) are unknown.

## Introduction

The Hood River basin, Oregon, is located along the southern edge of the Columbia River in the transition zone created by the Cascade Range between the western wet temperate and eastern dry continental climates. The basin’s unique geography and glacier-fed streams have made it home to one of the state’s most diverse assemblages of native anadromous and resident fish, including spring and fall Chinook salmon (*Oncorhynchus tshawytscha*), summer and winter steelhead (anadromous *O*. *mykiss*), coho salmon (*O*. *kisutch*), bull trout (*Salvelinus confluentus*), Pacific lamprey (*Entosphenus tridentatus*), rainbow trout (*O*. *mykiss*), cutthroat trout (*O*. *clarkii*), and mountain whitefish (*Prosopium williamsoni*) [1,2]. Five of the six anadromous populations (spring and fall Chinook, summer and winter steelhead, and coho) and one resident population (bull trout) are currently listed as threatened under the Endangered Species Act (ESA) due to population declines [2]. The spring Chinook salmon population in the Hood River was extirpated in the 1970s and reintroduction efforts using hatchery stock from the neighboring Deschutes River basin have been underway since 1993 [3,4]. Six hundred kilometers of streams in the Hood River basin are classified as critical habitat for salmonids and the basin is considered essential for recovery of several Lower Columbia Salmon and Steelhead Evolutionarily Significant Units, which are currently identified as being at high risk of extinction [5–7].

In-stream pesticide presence has been identified as a potential threat to salmonids in this primarily forested and agricultural basin and more broadly throughout the Pacific Northwest of North America. Since 1999, the Pesticide Stewardship Partnership program has engaged farmers, agricultural extension agents, watershed groups, tribes, and state agencies to reduce currently used pesticide presence in the basin’s streams [8]. The program: 1) helps pesticide users implement best management practices to reduce pesticide movement and harmful effects to non-target organisms, 2) measures pesticide presence in stream water samples collected and analyzed by Oregon Department of Environmental Quality (ODEQ), and 3) adapts watershed management practices based on the findings. Data from the late 1990s and early 2000s revealed the presence of organophosphate (OP) insecticides that had been commonly used on orchards, some at concentrations exceeding state water-quality criteria set to protect aquatic organisms [9,10]. A summary of pesticide concentrations measured in the basin from 1999–2009 identified some information gaps in the understanding of chemical contaminants and streams of concern for threatened salmonid fisheries [11], including contaminant presence in the basin throughout the year, contaminant presence in the upper basin and hydrophobic contaminant presence (pyrethroid insecticides, polybrominated diphenyl ethers [PBDEs], and polychlorinated biphenyls [PCBs], which have not previously been sampled).

This paper summarizes continuation of long-term pesticide monitoring in the basin in 2011–2012, with an emphasis on addressing temporal and analytical gaps identified previously [11]. Broad objectives are to: 1) characterize current in-stream pesticide presence and concentrations, 2) assess risks to salmonids in Hood River basin streams from pesticide exposure based on the available data, and 3) provide data with which to evaluate the outcomes of best management practices implemented in the basin. This work focuses on basin-wide contaminant threats to salmonid health and productivity, and complements the ongoing efforts of the Pesticide Stewardship Partnership, which assesses impacts on streams from various pesticide use activities. Specifically, this work provides time-weighted average (TWA) concentration data on pesticide mixtures in the basin to improve understanding of potential effects of ambient conditions on salmonids and their prey. The year-round use of passive integrative samplers to monitor pesticides in streams described here is a novel approach in surface-water-quality monitoring and investigation.

Passive sampling addresses some of the limitations inherent to discrete (grab) sampling, including the episodic presence of pesticides and the low concentrations at which they can be present [12–15]. Passive sampling also allows determination of TWA concentrations of pesticides, which represents chemical exposure for aquatic organisms [16]. Two types of passive samplers, polar organic chemical integrative samplers (POCIS) and semipermeable membrane devices (SPMDs), were used in this study. POCIS were used to sample for a broad suite of hydrophilic (water-soluble) pesticides, whereas SPMDs sampled hydrophobic (lipid-soluble) contaminants. SPMDs passively accumulate dissolved in-stream contaminants, mimicking the uptake of contaminants in the lipids of aquatic organisms, but unlike in biological systems, SPMDs do not metabolize accumulated compounds [13,17]. SPMDs provide a measure of bioavailable pollutants, which can provide a link to the risk of exposure to aquatic organisms. Although many of the chlorinated pesticides that SPMDs sample have been banned for several decades, these compounds tend to persistent in the environment, be highly toxic to organisms, and bioaccumulate.

## Materials and Methods

### Sampling sites

**Fig 1:**
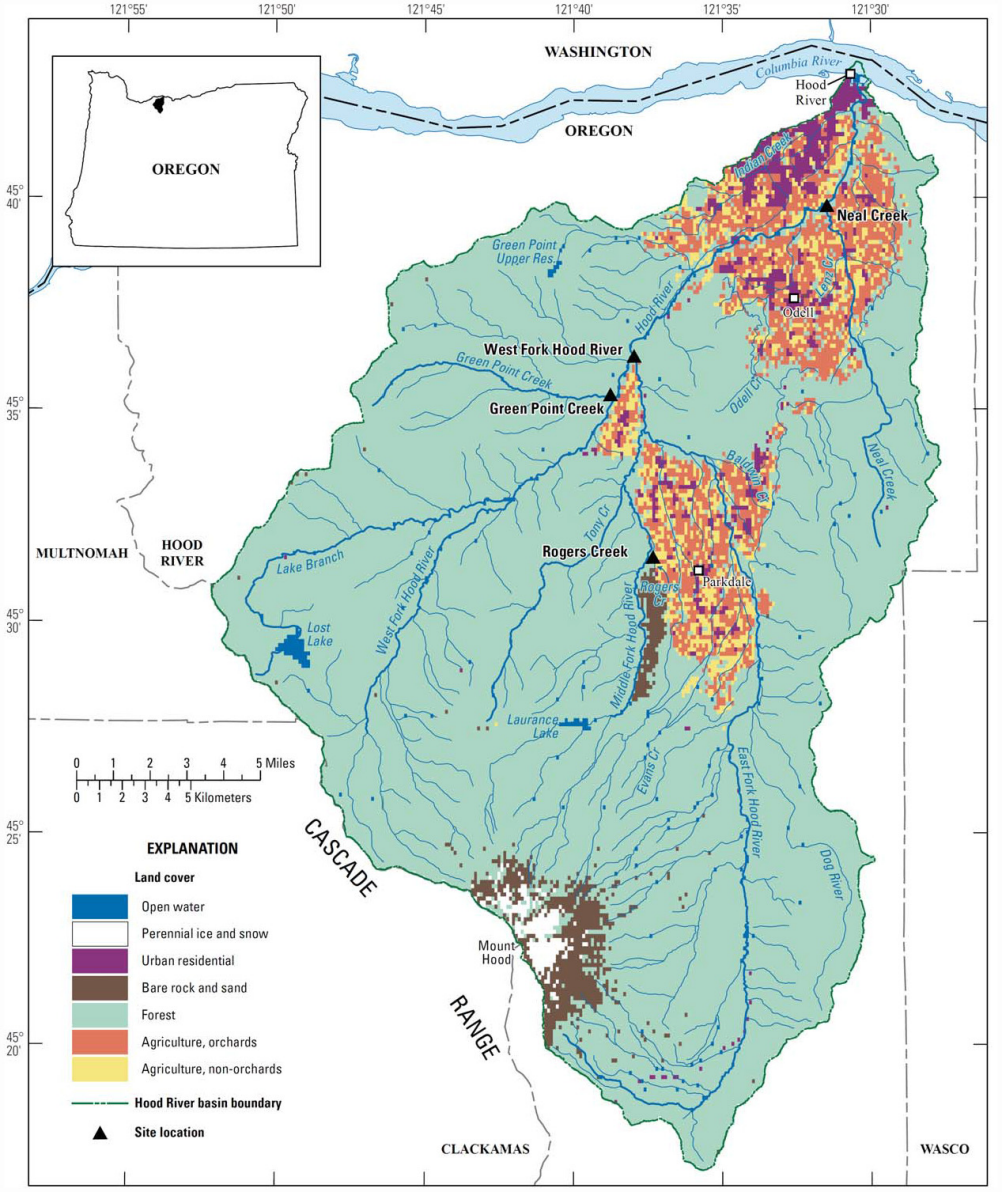
Map of Hood River basin, Oregon. Passive sampling sites are marked with triangles. The base map is modified from [11].

Sampling during 2011–2012 occurred at four sites in the Hood River basin: Neal Creek, Rogers Creek, Green Point Creek, and West Fork Hood River (Fig 1). Sampler deployments lasted 2 months each (Table 1). Two sites, Neal Creek and Rogers Creek, were sampled year-round over six deployments. Monitoring at these sites was intended to address the temporal gaps in the existing dataset identified elsewhere [11]; very few fall or winter samples were previously collected. The two upper watershed sites, Green Point Creek and West Fork Hood River, were monitored near their mouths during a single deployment in the fall, when pesticide use is known to occur on forested lands and overland runoff to streams is expected. Samplers were placed within the stream channels at points where they would be: 1) in the path of continuously flowing water, 2) submerged throughout the deployment, and 3) at minimal risk of vandalism. Samplers were attached to metal stakes and suspended off the sediments at mid-depth in the water column.

**Table 1:** Deployment dates of passive samplers, Hood River basin, 2011–2012.

Deployment period	Dates deployed	Number of days deployed	Season
Neal Creek and Rogers Creek			
1	3/11/2011–5/13/2011	66	Spring
2	5/13/2011–7/13/2011	61	Summer
3	7/13/2011–9/14/2011	63	Summer
4	9/14/2011–11/14/2011	61	Fall
5	11/14/2011–1/16/2012	63	Winter
6	1/16/2012–3/14/2012	58	Winter
Green Point Creek and West Fork Hood River			
7	8/26/2011–10/25/2011	62	Summer/Fall

#### Year-round sampling sites

Neal Creek and Rogers Creek were monitored continuously during Mar. 2011–2012 to determine the year-round presence of pesticides. The Neal Creek site “Neal Creek at the mouth” [11] was sampled every year since 1999 by ODEQ using discrete sampling techniques, typically 15–20 times annually in the spring and early summer (Mar.–June) and fall (Sept.–Oct.). Fourteen of the 19 pesticides that were detected in the basin in 1999–2009 were detected in Neal Creek, and many of the highest concentrations detected were in Neal Creek or its tributary Lenz Creek, which receives pesticide-laden fruit processing facility wastewater discharge [11]. Neal Creek provides important habitat for winter and summer steelhead and coho salmon [7]. Major land cover classifications in the Neal watershed are forest (75%) and agriculture (23%). The Neal Creek site was accessed with permission from the private landowner. Rogers Creek is a tributary to the Middle Fork Hood River that was sampled intermittently in 2008–2010 by ODEQ. The Parkdale Fish Facility, located on Rogers Creek, is an important facility for spawning, incubation, and release of spring Chinook and winter steelhead to Rogers Creek and other streams throughout the basin [18]. Major land cover classifications in the Rogers watershed are lava flow (bare rock) (76%), agriculture (14%), and forest (9%). The Rogers Creek site was accessed through an easement with the private landowner granting permission for monitoring and hatchery maintenance.

#### Upper watershed sites

Green Point Creek and West Fork Hood River, two streams in the upper watershed, were sampled near their mouths during fall 2011. These sites were selected in coordination with local foresters in order to target areas that would be harvested for timber and sprayed during the passive sampler deployment. Green Point Creek is a tributary to the West Fork Hood River that has important summer and winter steelhead, coho salmon, and rainbow trout habitats [7] and has not been previously sampled for pesticides. The Green Point Creek site was accessed with permission from the private landowner. West Fork Hood River is an important release point for hatchery-reared Chinook salmon smolts into the Hood River basin [3,19] and provides habitat for summer and winter steelhead, spring Chinook, and coho salmon [7]. The West Fork Hood River monitoring site was sampled by ODEQ in 2008–2009 and was accessed via a public road and by wading through this state-owned waterbody. The watersheds upstream of both sites are more than 95% forested.

### Sampling and analytical methods

Passive samplers (POCIS and SPMDs) were simultaneously deployed at sites in the Hood River basin during Mar. 2011–Mar. 2012. A large range of contaminants is measured through concurrent use of the two samplers [13]. At all sites, SPMDs and POCIS were deployed simultaneously in preloaded deployment canisters provided by the USGS Columbia Environmental Research Center (CERC). Each canister contained four POCIS and one SPMD. PCB congeners 14, 29, and 50 were added to the SPMDs and used as performance reference compounds (PRCs), because they do not occur in the environment [13]. PRCs are chemicals that have a moderate to high potential to dissipate from the sampler, which are added during fabrication and are used to account for the effects of stream flow, temperature, and biofilm accumulation on the sampler’s surface when calculating water concentrations [13].

After the deployment period ended, water concentrations were estimated from concentrations of contaminants of interest in the SPMD. In this study, SPMDs were analyzed for chlorinated pesticides, selected PBDE congeners, total PCB congeners, and PRCs at the USGS CERC laboratory using methods detailed elsewhere [13,20,21]. The chemicals were recovered from the SPMD through a dialysis process. The samples then underwent a series of cleanup and fractionation processes including size exclusion chromatography and gravity-flow chromatography using Florisil^®^ and silica gel to isolate the chemicals of interest from potential interferences. A list of compounds sampled using SPMDs is provided in Table A in S1 Table.

A POCIS samples hydrophilic contaminants and can be used to estimate average exposure to polar organic chemicals over time [22]. Chemicals of interest were recovered from the POCIS using a methanol solvent extraction. Extracts from two POCIS from each site were combined into a single sample in order to increase the amount of chemical available for detection. Samples were analyzed for currently used (CU) insecticides and fungicides using gas chromatography/mass spectrometry at the USGS California Water Science Center using methods detailed elsewhere [23]. The extracts from the remaining two POCIS from each site were combined into a single sample and analyzed for a suite of herbicides using liquid chromatography/diode array detection at the USGS CERC laboratory [24]. A list of compounds sampled using POCIS is provided in Tables B and C in S1 Table. Compounds listed in Tables A and B in S1 Table were analyzed at all sites and deployments. Additionally, 10 herbicides used in forestry were analyzed from the deployment at the two upper watershed sites only (Table C in S1 Table).

### Quality assurance

Blanks and recovery spikes were used to ensure the reliability of the measurements from the field. Blank samples included fabrication blanks, field blanks, and laboratory blanks as described by Alvarez [13]. Two fabrication blanks were created, one for the year-round sampling and one for the seasonal upper-watershed sampling. One field blank per site for each deployment was used during the year-round sampling (n = 12) and for the seasonal upper-watershed sampling (n = 2). No pesticides were detected in POCIS blank samples (Table A in S2 Table). Three forestry herbicides that were analyzed only in the upper-watershed sampling were detected in the POCIS fabrication and field blanks for Deployment 7 (Table B in S2 Table). Blank detections for the SPMDs are listed in Table C in S2 Table. Method detection limits (MDLs) were calculated as the mean of the blank detections plus three times the standard deviation of the blank detections for each chemical [13,25]. Method quantitation limits (MQLs) were calculated as the mean plus 10 times the standard deviation of the blank detections for each chemical. For analytes with no blank detections, the MDL was set at 20% of the lowest instrumental calibration standard and the MQL was set at the lowest instrument calibration standard (Tables A–C in S1 Table). Matrix and procedural spikes were performed as described by Alvarez [13]. The mean and standard deviation of triplicate spike recoveries for each compound analyzed from SPMDs are listed in Table F in S2 Table (mean ranges: 33–171% for organochlorine [OC] pesticides excluding chlorpyrifos at 345%, 66% for total PCBs, and 54–84% for PBDEs). Mean percent recoveries of d10-atrazine surrogate added to POCIS extracts were 98% (range: 79–118%) for the 14 field samples and 93% (76–116%) for the 14 field blanks (Table D in S2 Table). Mean percent recoveries of forestry herbicides added to POCIS extracts ranged from 92 to 105% for the 10 analyzed pesticides (Table E in S2 Table). Reported water concentrations from the passive samplers were not recovery corrected.

### Comparison to discrete sample data

The integration of contaminants from pulsing releases (such as after runoff events) collected over a longer time period makes passive sampling data more representative of long-term exposures compared to data from discrete sampling [15]. However, TWA concentrations can dampen shorter term peaks that could be detected in well-timed discrete sampling. Comparisons of passive sampler data to discrete measurements is limited as in many cases the concentrations measured in a passive sampler are below the detection limits of what is obtainable in a single 1-liter discrete water sample. A review of several studies indicates that TWA concentrations from SPMDs generally agree within a factor of 3 to measured concentrations in discrete samples [26]. For a more detailed review of the comparisons between passive and discrete sample data, see S2 Text.

### Reporting of data

The concentrations of chemicals measured in POCIS and SPMDs were used to estimate average water concentrations using experimentally derived chemical sampling rates [13]. Water concentrations of compounds from both samplers are reported in nanograms per liter (ng/L). For four analytes without established chemical sampling rates, results are reported as mass of the chemical residue per POCIS (ng/POCIS). In such a case, the actual water concentration is not known, but the results indicate the presence or absence of those chemicals during a deployment [13].

Detected compounds are classified as either currently used (CU) or legacy. Currently used pesticides (CUPs) have approved registrations for use from the U.S. Environmental Protection Agency (EPA). Legacy compounds are no longer used (or their use is severely restricted) but are still detected in environmental media due to their persistence for years to decades in the environment, most notably in soils, sediments, and organisms. All legacy compounds in this study are hydrophobic, while the CUP category includes hydrophobic and hydrophilic pesticides. Some compounds, such as PBDEs and recently banned pesticides, appear in the literature in both categories (CU and legacy compounds). Here, PBDEs are considered legacy because their use was banned in Oregon by the time of data collection, whereas endosulfan is considered a CUP because it was in use in orchard crops at that time, although those uses have since been phased out (the last uses, on vegetable crops for seed and strawberries, will be phased out in July 2016) [27].

### Risk assessments

Detected concentrations were compared to U.S. EPA Office of Pesticide Programs aquatic life benchmarks [28], U.S. EPA water-quality criteria [29] and ODEQ water-quality criteria [30], which are set for the protection of organisms, and the U.S. EPA water-quality criteria for human health [31]. Acute thresholds are appropriate for comparison to environmental samples collected over a short time scale (up to 1 day). Chronic thresholds are appropriate for comparison to samples collected over a longer time scale (4 to 60 days). TWA concentrations were compared to chronic thresholds, whereas discrete sample concentrations collected by ODEQ during 2011 and 2012 [32,33] were compared to acute thresholds. Detected concentrations were also compared to median lethal concentrations (LC50s) and those from the literature shown to cause sublethal effects to aquatic organisms. Although LC50s are based on 48- or 96-hour exposures, they are used as conservative screening levels here; if TWA concentrations are larger than the LC50s, then short-term concentrations definitely exceeded those thresholds, but their duration and frequency cannot be determined using passive sampling data.

The Sensitive Pesticide Toxicity Index (SPTI) approach [34] was applied to the detected pesticide concentrations from Neal and Rogers Creeks and ODEQ grab samples as a screening tool with which to assess relative toxicity among samples. The approach uses a concentration-addition model, summing the ratios of detected concentrations of pesticides to the 5th percentile of the LC50 values (or minimum value, if there were insufficient data to compute a 5th percentile) from the literature for each pesticide toward two target groups of organisms, fish and benthic invertebrates. The sum of those ratios for each sample is its SPTI value. This approach provides a more conservative indicator of toxicity than the standard PTI approach, which uses median LC50 values, and is therefore appropriate for sensitive species and life stages [34], which are present in the basin. Its application with TWA concentrations is additionally conservative because it assumes that all of the compounds detected in a sample were present at the same time. Each passive sampler deployment in each stream was considered as a separate sample. Sensitive toxicity concentrations for fish and benthic invertebrates came from Tables B.1 and B.3 (supplemental materials in [34]). In order to make the indices as comprehensive as possible, LC50 values for pesticides not included in that source [34] were taken from other summaries of the literature [35–37] or from primary references if summaries were not available. It is unlikely that the described approach biased the results, as the dominant contributors to the overall toxicity of mixtures were pesticides that were included in the original dataset [34]. For the SPTIs, compounds detected at concentrations less than the MQL were assigned concentrations equal to the MDL for that compound (S1 Table). However, the indices exclude concentrations of pesticides for which water concentrations could not be calculated (those reported in ng/POCIS) and PBDEs, for which there are not LC50 values in the literature. Toxicity data used in the SPTI calculations are in S4 Table.

## Results and Discussion

### Summary of detections at year-round sites

Six of the 98 pesticides analyzed from the POCIS samplers and representatives of all analyzed classes of contaminants in SPMDs except for PCBs were detected in Neal and Rogers Creeks (S1 Table). Hexazinone and simazine, herbicides previously detected in the basin, the insecticide endosulfan and its degradates and the fungicide pyrimethanil were detected during all six deployments at Neal Creek (Table 2), as were the legacy OC pesticides or degradates, DDTs, dieldrin, hexachlorobenzene (HCB), and pentachloroanisole (PCA) (Table 3). Pyrethroid insecticides were detected during two deployments and PBDEs were present in five deployments in Neal Creek.

**Table 2:** Time-weighted average concentrations of currently used pesticides detected in passive samplers, Hood River basin, Oregon, 2011–12.

	Neal Creek						Rogers Creek						Green Point Creek	West Fork Hood River
Deployment period	1	2	3	4	5	6	1	2	3	4	5	6	7	7
Compound	Mar. 11–May 13, 2011	May 13–July 13, 2011	July 13–Sept. 14, 2011	Sept. 14–Nov. 14, 2011	Nov 14, 2011–Jan. 16, 2012	Jan. 16–Mar. 14, 2012	Mar. 11–May 13, 2011	May 13–July 13, 2011	July 13–Sept. 14, 2011	Sept. 14–Nov. 14, 2011	Nov 14, 2011–Jan. 16, 2012	Jan. 16–Mar. 14, 2012	Aug. 26–Oct. 25, 2011	Aug. 26–Oct. 25, 2011
Concentrations of compounds in water, in nanograms per liter														
Currently Used Herbicides														
Hexazinone	15	14	5.6	6.9	8.1	14	2.9	2.9	--	--	--	--	--	--
Simazine	21	36	19	12	16	16	--	2.8	--	--	--	--	--	--
Trifluralin	*Present*	*Present*	--	*Present*	*Present*	--	--	*Present*	*Present*	*Present*	*Present*	--	--	*Present*
2,4-D	na	na	na	na	na	na	na	na	na	na	na	na	170	250
Chlorsulfuron	na	na	na	na	na	na	na	na	na	na	na	na	27	26
Metsulfuron methyl	na	na	na	na	na	na	na	na	na	na	na	na	--	70
Sum of Herbicides	36	50	24	19	24	30	3	6	*Present*	*Present*	*Present*	--	197	346
Currently Used Insecticides and Degradates														
Endosulfans														
Endosulfan	5.40	1.10	1.20	0.59	0.85	12.0	--	--	--	--	--	--	--	*Present*
Endosulfan-II	0.53	4.80	7.80	4.30	3.30	9.40	*Present*	*Present*	--	--	--	--	--	*Present*
Endosulfan Sulfate	3.10	4.40	9.50	4.10	3.90	2.80	*Present*	*Present*	--	*Present*	*Present*	*Present*	*Present*	*Present*
Sum of Endosulfans	9.03	10.3	18.5	8.99	8.05	24.2	*Present*	*Present*	--	*Present*	*Present*	*Present*	*Present*	*Present*
Pyrethroids														
Total Cyfluthrins	--	0.140	--	--	--	--	--	0.130	*Present*	--	--	--	--	--
Total Cypermethrins	--	--	--	--	*Present*	--	--	--	--	--	--	--	--	--
Esfenvalerate	--	--	--	--	*Present*	--	--	--	--	--	--	--	--	--
Deltamethrin	--	*Present*	--	--	--	--	--	--	--	--	--	--	--	--
Sum of Pyrethroids	--	0.140	--	--	*Present*	--	--	0.130	*Present*	--	--	--	--	--
Organophosphates														
Chlorpyrifos	0.200	*Present*	*Present*	*Present*	*Present*	*Present*	--	--	--	--	--	--	--	--
Currently Used Fungicides														
Pyrimethanil	3.6	5.5	2.8	15	15	6.4	--	--	--	--	--	--	--	--
Concentrations of compounds per POCIS, in nanograms per POCIS														
Currently Used Herbicides and Degradates														
3,4-DCA	38	190	--	--	--	--	--	--	--	--	--	--	--	--
Triclopyr	na	na	na	na	na	na	na	na	na	na	na	na	170	250
Currently Used Fungicides and Degradates														
Boscalid	60	170	180	77	84	100	--	*Present*	*Present*	--	--	--	--	--
3,5-DCA	43	220	20	--	23	--	--	--	--	--	--	--	--	--

Concentrations in water were estimated using compound-specific sampling rates. Compounds without sampling rates are presented in concentrations per POCIS. See Tables A and B in S1 Table for complete list of compounds analyzed in the SPMDs and POCIS, respectively. [na, not analyzed; --, not detected; *Present*, presence is greater than the method detection limit, but concentration is not quantified because it is less than the method quantitation limit]

**Table 3:** Time-weighted average concentrations of legacy compounds detected in passive samplers, Hood River basin, Oregon, 2011–12.

	Neal Creek						Rogers Creek						Green Point Creek	West Fork Hood River
Deployment period	1	2	3	4	5	6	1	2	3	4	5	6	7	7
Compound	Mar. 11–May 13, 2011	May 13–July 13, 2011	July 13–Sept. 14, 2011	Sept. 14–Nov. 14, 2011	Nov 14, 2011–Jan. 16, 2012	Jan. 16–Mar. 14, 2012	Mar. 11–May 13, 2011	May 13–July 13, 2011	July 13–Sept. 14, 2011	Sept. 14–Nov. 14, 2011	Nov 14, 2011–Jan. 16, 2012	Jan. 16–Mar. 14, 2012	Aug. 26–Oct. 25, 2011	Aug. 26–Oct. 25, 2011
Concentrations of compounds in water, in nanograms per liter														
Legacy Insecticides and Their Degradates														
DDTs														
*o*,*p'*-DDD	0.040	0.052	0.077	0.049	0.040	0.026	--	--	--	--	--	--	--	*Present*
*p*,*p'*-DDD	0.110	0.170	0.340	0.200	0.130	0.073	*Present*	0.013	0.009	0.010	*Present*	*Present*	--	--
*o*,*p'*-DDE	*Present*	0.009	0.009	0.007	0.007	*Present*	--	--	--	--	--	--	--	--
*p*,*p'*-DDE	0.610	0.940	1.000	0.680	0.470	0.610	0.027	0.028	0.028	0.037	0.031	0.035	--	*Present*
*o*,*p'*-DDT	0.036	0.039	0.039	0.025	0.025	0.031	*Present*	*Present*	*Present*	*Present*	*Present*	*Present*	--	--
*p*,*p'*-DDT	0.250	0.287	0.326	0.165	0.160	0.214	0.015	0.012	0.010	0.011	0.009	0.015	*Present*	0.007
Sum of DDTs	1.046	1.496	1.790	1.126	0.833	0.954	0.042	0.053	0.047	0.059	0.040	0.050	*Present*	0.007
Chlordanes														
cis-Chlordane	--	--	--	--	--	--	--	--	--	--	--	--	--	*Present*
trans-Chlordane	*Present*	*Present*	*Present*	*Present*	*Present*	*Present*	--	--	--	--	--	--	--	*Present*
Oxychlordane	*Present*	*Present*	*Present*	*Present*	*Present*	*Present*	--	--	--	--	--	--	--	*Present*
cis-Nonachlor	--	*Present*	--	*Present*	--	--	--	*Present*	--	*Present*	--	--	--	--
trans-Nonachlor	*Present*	0.008	*Present*	*Present*	--	*Present*	--	--	--	--	--	--	--	*Present*
Sum of Chlordanes	*Present*	0.008	*Present*	*Present*	*Present*	*Present*	--	*Present*	--	*Present*	--	--	--	*Present*
Other Organochlorine Insecticides and Degradates														
Dieldrin	0.089	0.120	0.310	0.130	0.120	0.081	--	--	--	--	--	--	--	--
Endrin	*Present*	*Present*	*Present*	*Present*	*Present*	*Present*	--	--	--	--	--	--	--	--
Heptachlor Epoxide	*Present*	*Present*	*Present*	*Present*	*Present*	*Present*	--	--	--	*Present*	--	--	--	*Present*
Lindane	--	--	--	--	--	*Present*	--	--	--	--	--	--	--	--
*p*,*p'*-Methoxychlor	*Present*	--	--	--	*Present*	*Present*	--	--	--	--	--	--	--	--
Legacy Fungicides and Degradates														
Hexachlorobenzene (HCB)	0.011	0.012	0.010	0.009	0.010	0.012	*Present*	*Present*	*Present*	*Present*	*Present*	*Present*	0.015	0.013
Pentachloroanisole (PCA)	*Present*	0.052	0.044	0.059	0.077	0.055	--	--	--	--	--	--	--	*Present*
Brominated Flame Retardants														
PBDEs														
PBDE-28	*Present*	0.003	*Present*	*Present*	*Present*	--	--	*Present*	*Present*	--	--	--	--	--
PBDE-47	--	0.0002	--	--	--	--	--	0.190	*Present*	--	--	--	--	--
PBDE-66	--	*Present*	--	*Present*	--	--	--	--	--	--	--	--	--	--
PBDE-85	--	*Present*	--	--	*Present*	--	--	*Present*	--	--	--	--	--	--
PBDE-99	--	0.230	--	--	--	--	--	0.230	*Present*	--	--	--	--	--
PBDE-100	--	0.055	--	--	--	--	--	0.053	*Present*	--	--	--	--	--
PBDE-153	--	*Present*	--	--	--	--	--	*Present*	--	--	--	--	--	--
PBDE-154	--	0.017	--	--	*Present*	--	--	0.016	*Present*	--	--	--	--	--
Sum of PBDEs	*Present*	0.304	*Present*	*Present*	*Present*	--	--	0.489	*Present*	--	--	--	--	--

Compound-specific sampling rates were used to estimate water concentrations for all legacy compounds. See Tables A and B in S1 Table for complete list of compounds analyzed in the SPMDs and POCIS, respectively. [--, not detected; *Present*, presence is greater than the method detection limit, but concentration is not quantified because it is less than the method quantitation limit]

Among CUPs, only hexazinone, simazine, and cyfluthrin were detected at quantifiable concentrations in Rogers Creek. The total concentrations of herbicides were lower in Rogers Creek (2.8–2.9 ng/L) than in Neal Creek (5.6–36 ng/L). The pyrethroid insecticide cyfluthrin was detected at similar concentrations in Neal and Rogers Creeks (0.140 and 0.130 ng/L, respectively). DDTs were the only quantifiable legacy pesticides detected during all deployments in Rogers Creek. Concentrations of DDTs in Rogers Creek were similar among all deployments (0.040–0.059 ng/L). Concentrations of PBDEs were quantifiable only during one deployment in Rogers Creek and were similar to those in Neal Creek. PBDE-99 had the highest concentration among PBDE congeners in both streams (0.230 ng/L) and PBDE-47 was nearly as high in Rogers Creek (0.190 ng/L). These very stable congeners are frequently detected in the environment and organisms in the Columbia Basin [38–42]. Although commonly detected at low levels in the environment [43], PBDE-183 was not detected in this study.

### Temporal detection patterns at year-round sites

**Fig 2:**
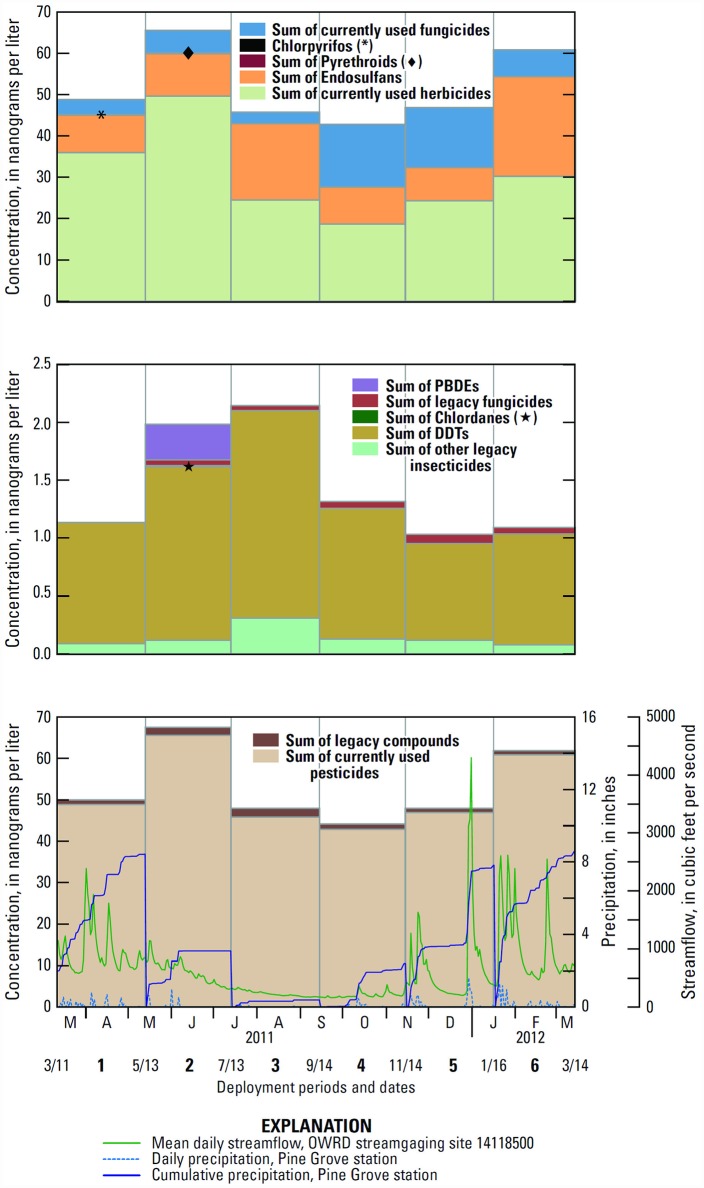
Compounds detected in passive samplers in Neal Creek Oregon, 2011–2012: (A) currently used pesticides; (B) legacy compounds;(C) all compounds and seasonal precipitation and streamflow. [44,45].

Compound detections in Neal Creek differed across deployments (Fig 2). Summed CUP concentrations in water in Neal Creek were similar among deployments 1, 3, 4, and 5 (43–49 ng/L). Deployment 2 had the highest summed CUP concentrations (66 ng/L, May–July), primarily because of the high simazine concentration (36 ng/L) and Deployment 6 (61 ng/L, Jan.–Mar. 2012), primarily due to the peak endosulfan concentration (24 ng/L).

**Fig 3:**
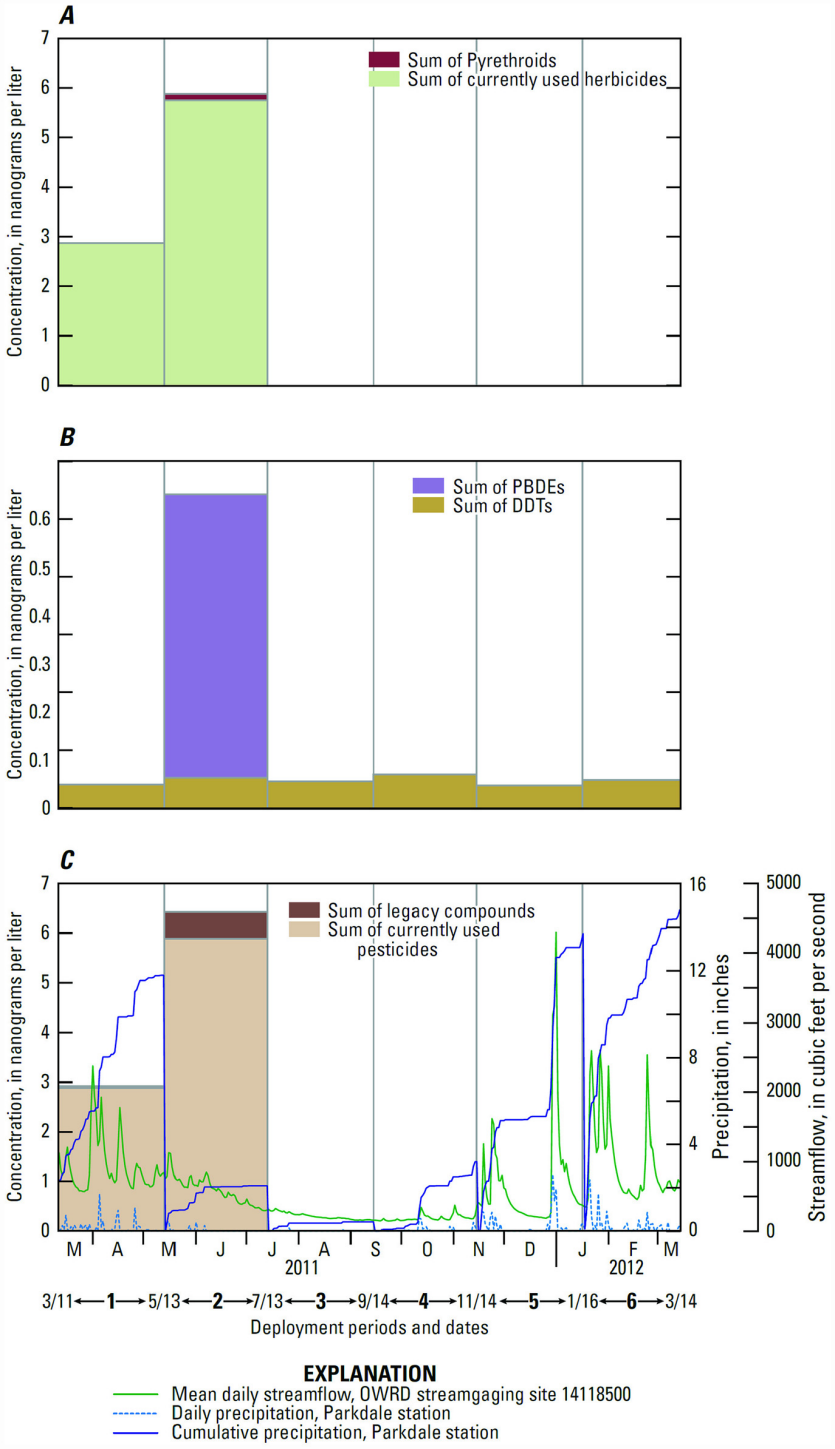
Compounds detected in passive samplers in Rogers Creek Oregon, 2011–2012: (A) currently used pesticides; (B) legacy compounds; (C) all compounds and seasonal precipitation and streamflow. [44,45].

The total concentration of CUPs in Rogers Creek was highest during the May–July deployment (5.9 ng/L) mainly due to the detections of hexazinone (2.9 ng/L) and simazine (2.8 ng/L), followed by the Mar.–May deployment (2.9 ng/L) due to the detection of hexazinone (2.9 ng/L) (Fig 3). Detections of CUPs in Rogers Creek occurred during their expected timing of potential use in the basin (Table 4).

**Table 4:** Use, seasons of detection and expected application, persistence, and mobility of currently used pesticides detected in the Hood River basin, Oregon, 2011–2012.

Compound	Type	Potential uses	Expected season of use	Season of detection	Indicators of Persistence		Indicator of Mobility
					Half-life in water, in days	Half-life in anaerobic soil, in days	log Koc
Chlorsulfuron	H	forestry; ROW	late winter, spring	summer, fall	1230	28	1.54
2,4-D	H	forestry; ROW	spring, fall	summer, fall	39	34	1.67
Triclopyr	H	forestry; ROW	late spring	summer, fall	8.7	39	1.68
Metsulfuron methyl	H	forestry	spring, fall	summer, fall	30	24	1.76
Pyrimethanil	F	orchard crops, wine grapes	spring, summer, fall	all	unknown	55	2.47
Simazine	H	orchard crops, grapes, blueberries, forestry, ROW	spring, fall	all	28	110	2.53
3,4-DCA	D (H)	(parent compound, diuron): orchard crops, ROW	spring, fall (diuron)	spring, summer	1290 (diuron)	372 (diuron)	2.70
Hexazinone	H	forestry, ROW, blueberries	spring	all	56	222	2.81
3,5-DCA	D (F)	(parent compound, iprodione): orchard crops	spring, summer (iprodione)	spring, summer	no data	no data	2.49
Boscalid	F	orchard crops	spring, summer	all	30	347	2.88
Chlorpyrifos	I	orchard crops	late winter, early spring	spring, *summer*, *fall*, *winter*	2118	31	4.00
Endosulfan	I	orchard crops	early spring	all	no data	27	4.09
Cyfluthrin	I	orchard crops, residential uses	late spring, summer	summer	215	33	4.80

Only compounds that were detected at concentrations greater than the method quantitation limit are included here. [D, degradate; F, fungicide; H, herbicide; I; insecticide; Koc, organic carbon-water partitioning coefficient; ROW, rights-of-way; italicized season of detection indicates that the compound was present at levels that were too low to be quantified.] (Information on potential pesticide uses and timing came from the personal communications with: Anne Saxby, Hood River Soil and Water Conservation District and Steve Castagnoli, Oregon State University Extension Service; and from [46,47]. Persistence and mobility information came from [37,46,48])

### Pesticides at upper watershed sites

Four herbicides that are used in forests or for rights-of-way were detected in the single POCIS deployment in West Fork Hood River and Green Point Creek during late Aug.–Oct. 2011: triclopyr, 2,4-D, chlorsulfuron, and metsulfuron methyl (Table 2). Concentrations of those herbicides were equal or nearly equal in the two streams, except that metsulfuron methyl was detected in West Fork Hood River only. CU insecticides (e.g., pyrethroids and chlorpyrifos) were analyzed but not detected at these sites. The only quantified concentrations of legacy OC pesticides were of HCB at both sites and *p*,*p’*-DDT at West Fork Hood River only (Table 3).

### Comparison to discrete sample data

**Fig 4:**
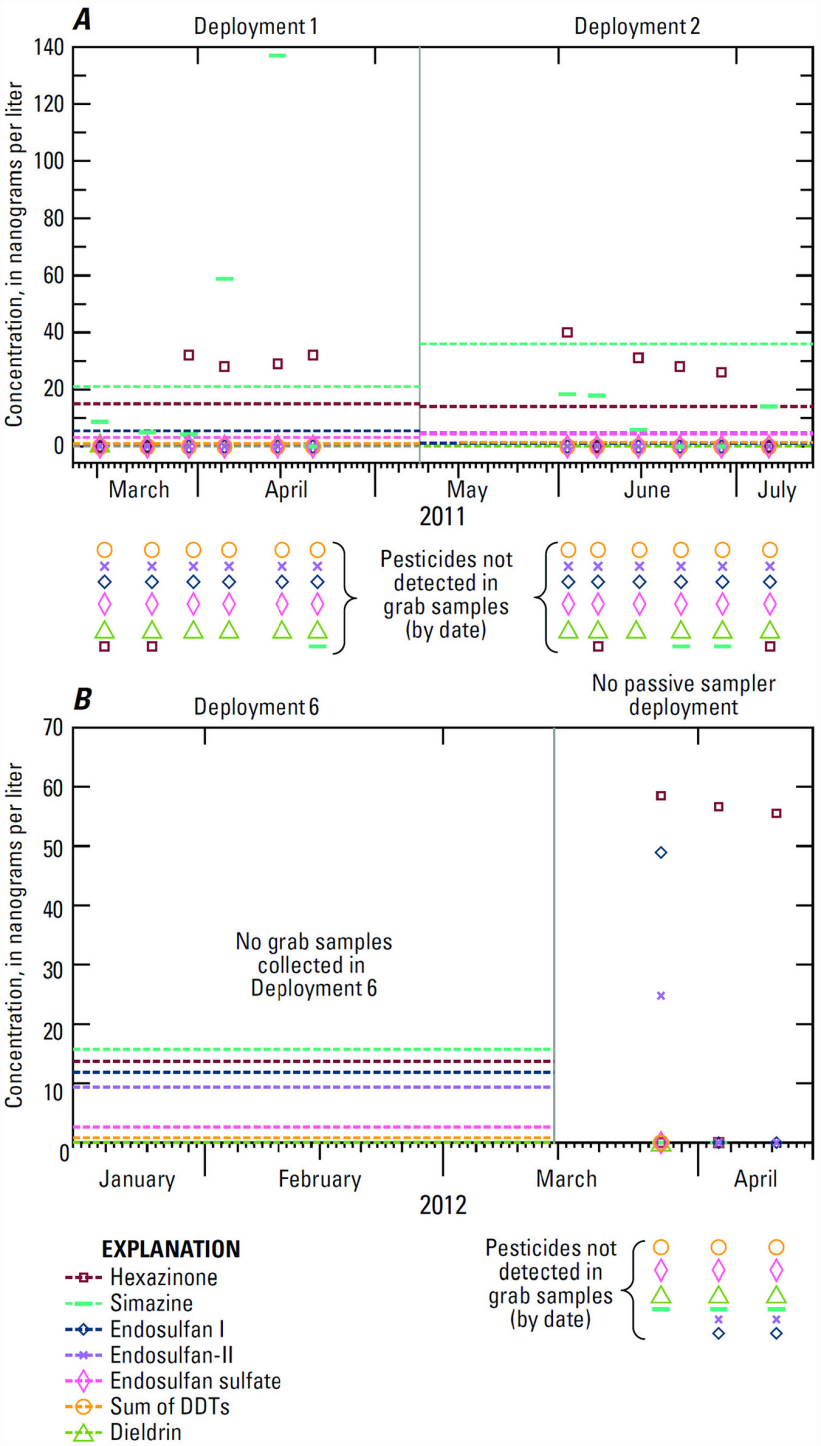
Concentrations of detected pesticides from passive and discrete sampling: (A) during Deployments 1 and 2 (Mar.–July, 2011); (B) during and 1 month after Deployment 6 (Jan.–Apr., 2012). Horizontal lines show time-weighted average concentrations from passive samplers; symbols show concentrations from discrete (grab) samples collected by Oregon Department of Environmental Quality [32].

Peak ODEQ grab sample concentrations exceeded the TWA concentrations for hexazinone and simazine, the only two compounds detected from both sampling approaches in Mar.–July 2011 (Fig 4A). Several compounds were detected in passive but not grab samples collected during those deployments, mostly hydrophobic pesticides that are difficult to detect in traditional grab samples. S1 Table indicates which compounds were analyzed with both approaches. Occasional pulses can result in TWA concentrations that are less than the passive sampler detection limit for a compound, as appears to have been the case for carbaryl, which was detected in two ODEQ grab samples (27.4 and 8 ng/L, Table A in S3 Table) in early June and July 2011 [32], but was not detected in the POCIS deployed from mid-May to mid-July.

The ODEQ discrete sampling in 2012 began 2 weeks after the end of the last passive sampler deployment. Endosulfan-I and -II were detected in grab samples from Neal Creek in Mar. 2012 (Table B in S3 Table) (48.9 and 24.7 ng/L, respectively) for the first time since being added to the analyte list in 2009 [33] and following the passive sampling deployment with the highest endosulfan concentrations (Fig 4B). Diuron, hexazinone, simazine, and imazapyr were detected from most grab samples collected from Neal Creek in spring 2012, but at very low concentrations. Carbaryl was detected in Neal Creek in May 2012 (82.9 ng/L). Chlorpyrifos was not detected in Neal Creek, but was detected in Odell Creek in Apr. 2012 (31.4 ng/L), during the same time of year as it was detected in passive samplers in Neal Creek in 2011 (TWA 0.2 ng/L). Malathion, the only other OP insecticide detected in 2012 grab samples, was detected in Odell Creek in June 2012 (135 ng/L).

### Potential sources, fate, and transport of detected compounds

#### Currently used pesticides (CUPs)

CUPs for agriculture, forestry, and rights-of-way pest control were detected in the POCIS and SPMDs. Table 4 shows timing of detection and potential use for the CUPs detected in passive samplers. Among the sites, Neal Creek has the largest proportion of orchard land in its drainage area, most of which is in the lower drainage area near the sampling site. All detected pesticides with potential orchard uses were detected in Neal Creek during all sampling deployments (Tables 2 and 4), except pyrethroids, which were detected only during two. In contrast, the same pesticides (pyrimethanil, simazine, boscalid, chlorpyrifos, endosulfan, and cyfluthrin) were detected intermittently or not at all in Rogers Creek, which has a relatively small proportion of upstream land in orchards. Pear orchards cover ~80% of the basin’s orchard acreage. Pesticides are typically applied to pear orchards in the mid-Columbia region during every month except Dec. and Jan. [46]. Forestry and rights-of-way herbicides are typically applied during spring and fall (Table 4). Pyrimethanil, simazine, hexazinone, boscalid, and endosulfan were detected year-round, even though their use is expected only during 1–2 seasons. The detected compounds are expected to persist in soils, surface water and/or groundwater for one month to several years based on their half-lives in those media. Water-soluble herbicides used in forestry (e.g., 2,4-D, chlorsulfuron, hexazinone, metsulfuron methyl) are more likely to be transported to streams in runoff, whereas sediment-bound pesticides, such as orchard insecticides (e.g., chlorpyrifos, endosulfan, pyrethroids) tend to sorb to sediments and are typically transported in eroding soils.

Spring 2011 had moderate precipitation (Figs 2 and 3) [45] and spring has peak seasonal agricultural, forestry, and rights-of-way pesticide use in the basin (Table 4). Streamflow data are not available for Neal or Rogers Creeks, so streamflow from the West Fork Hood River [44] is used here to exemplify typical streamflow patterns in the basin. The Jan.–Mar. 2012 deployment had the highest total precipitation and several of the largest precipitation events during the monitoring period [45]. The peak concentration of endosulfan in Neal Creek during Jan.–Mar. could have resulted from seasonal use and precipitation-driven erosion, which has been shown to wash OC pesticides, including endosulfan, into surface waters [49]. The lower concentrations and frequency of contaminant detection in Rogers Creek compared to Neal Creek are likely due to differences in upstream land cover [11]. In addition to surficial runoff, Neal Creek and its tributary Lenz Creek also receive several fruit packing facilities’ discharge waters, which have had much higher pesticide concentrations than streams [11].

#### Legacy compounds

Legacy compounds have been banned by the U.S. EPA due to their persistence and high toxicity to humans and other non-target organisms. Many legacy compounds are hydrophobic, lipophilic, and bioaccumulate in organisms [50], including in high-lipid fish such as salmonids [51] and lampreys [52]. Legacy compounds detected in this study include several OC insecticides and fungicides, such as DDTs, trans-nonachlor, dieldrin, HCB, and PCA as well as PBDE flame retardants. Hydrophobic contaminants bind strongly to sediments, are typically transported into surface waters via contaminated sediment disturbance or soil erosion, and tend to accumulate in benthic areas that are used as salmonid habitat [53,54]. The higher total legacy contaminant concentrations in Neal Creek during deployments 2 and 3 (May–Sept.) compared to other deployments are mainly due to peak concentrations of DDTs. This peak could be from runoff of contaminated soils during the irrigation season (Apr. 15–Sept. 30) [55], as has occurred in Washington’s orchard-covered Yakima Basin [49]. Ratios of DDT isomers detected in all deployments in Neal and Rogers Creeks indicate that DDT was recently mobilized into the hydrologic system, for example, through erosion of contaminated soil [49].

Other contaminant transport pathways also exist; chlordane is commonly transported through atmospheric deposition [56] and PCP leaches from fungicide-treated lumber utility poles [57]. These chlorinated pesticides were widely used in the basin for agricultural, home, and public health pest control or as wood preservatives during the early- and mid-twentieth century, but most approved uses were banned during the 1970s and 1980s. Although HCB has no currently approved commercial uses in the United States, it is a byproduct of the manufacture of other pesticides and solvents and is sometimes a contaminant in other pesticides [58].

PBDE flame retardants are of concern due to their environmental persistence, continued entry into the environment via consumer and industrial products, and bioaccumulative potential [59]. They can cause toxicity and sublethal effects to fish at low concentrations [60]. PBDEs were frequently used in a variety of industrial and consumer goods (e.g., plastics, textiles, furniture, electronics), and their primary pathway to surface waters is thought to be laundry and wastewater effluent via household dust [59,61]. Products containing more than 0.1% of the penta- and octa- formulations were banned in Oregon in 2005 and deca formulations were banned in 2009 [62]. PBDEs were detected in wastewater treatment effluent in Hood River in Dec. 2008 [41] and in effluent from 51 of Oregon’s 52 major municipal wastewater treatment facilities in 2010 [63] and it is likely that they will continue to be delivered to surface waters via wastewater effluent due to the persistence of many PBDE-containing products in homes and businesses. Three penta-PBDEs, PBDE-85, -99, and -100 were detected in Neal and Rogers Creeks in this study. PBDEs are widely detected in the environment and can be transported far beyond their regions of use, as evidenced by their detections in multiple arctic species [64]. Atmospheric deposition from Asia, where handling and disposal of international electronic waste is a major source of PBDEs in the environment, is another source of PBDEs to North America’s Pacific coast [65,66]. Conversely, atmospheric deposition of pesticides in western U.S. national parks was attributed to regional sources rather than trans-Pacific transport [67].

### Comparisons to organism health thresholds

Oregon and federal water-quality standards and non-regulatory benchmarks are used here as screening thresholds with which to assess potential risks to organism health (Table 5) [28–30]. Endosulfan was the only compound detected at TWA concentrations exceeding any of these thresholds: the U.S. EPA benchmark for chronic exposure for invertebrates (10 ng/L, endosulfan-I+II) [28] was exceeded in Jan.–Mar. 2012 at Neal Creek. The benchmark was developed for comparison to 21-day average concentrations, so the use of 2-month TWA concentrations may underrepresent exceedances during 21-day periods. Although they were less than the lowest chronic thresholds set by the U.S. EPA and ODEQ, the maximum detected TWA concentrations in this study were within an order of magnitude of the thresholds for *p*,*p’*-DDT, endosulfan-II and endosulfan sulfate, so shorter-term concentrations could have been closer to the thresholds. All other maximum TWA concentrations in this study were at least 50 times less than lowest chronic-exposure thresholds for aquatic life [28–30].

**Table 5:** Maximum detected concentrations from passive samplers, Hood River basin, Oregon, 2011–12 compared to Oregon and U.S. EPA water-quality thresholds.

	U.S. EPA Office of Pesticide Programs Aquatic Life Benchmarks				U.S. EPA Water Quality Criteria		Oregon Water Quality Criteria		Oregon Aquatic Life Water Quality Guidance Values		Maximum Detected Time-Weighted Average Concentration	
	Fish		Invertebrates									
	Acute	Chronic	Acute	Chronic	CMC (Acute)	CCC (Chronic)	Acute	Chronic	Acute	Chronic	Concentration	Site and Deployment
**Benchmark averaging period**	individual sample	60 days	individual sample	21 days	24 hours	96 hours	1 hour	96 hours	1 hour	96 hours	na	na
**Currently Used Pesticides**												
2,4-D	--	--	12,500	--	--	--	--	--	--	--	0.25	WF Hood River, 7
Chlorpyrifos	0.9	0.57	0.05	0.04	0.083	0.041	0.083	0.041	--	--	0.0002	Neal Creek, 1
Chlorsulfuron	>150,000	32,000	>185,000	20,000	--	--	--	--	--	--	0.027	Green Point Creek, 7
Cyfluthrin	0.034	0.01	0.0125	0.0074	--	--	--	--	--	--	0.00014	Neal Creek, 2
Endosulfan, total (I+II)	0.05	0.11	0.3	**0.01**	--	--	0.22	0.056	--	--	0.0214	Neal Creek, 6
Endosulfan	--	--	--	--	--	--	0.22	0.056	--	--	0.012	Neal Creek, 6
Endosulfan-II	--	--	--	--	--	--	0.22	0.056	--	--	0.0094	Neal Creek, 6
Endosulfan sulfate	1.9		150		--	--	--	--	--	--	0.0095	Neal Creek, 3
Hexazinone	137,000	17,000	75,800	20,000	--	--	--	--	--	--	0.015	Neal Creek, 1
Metsulfuron methyl	>75,000	4,500	>75,000		--	--	--	--	--	--	0.07	WF Hood River, 7
Pyrimethanil	5,050	20	1,500	1000	--	--	--	--	--	--	0.015	Neal Creek, 5
Simazine	3,200		500		--	--	--	--	--	--	0.036	Neal Creek, 2
**Legacy Pesticides**												
Chlordanes, total	--	--	--	--	2.4	0.0043	2.4	0.0043	--	--	0.0000084	Neal Creek, 2
DDTs, total	--	--	--	--	--	--	--	--	--	--	0.001666	Neal Creek, 3
*p*,*p'*-DDD	--	--	--	--	--	--	--	--	0.06	--	0.00034	Neal Creek, 3
*p*,*p'*-DDE	--	--	--	--	--	--	--	--	1050	--	0.001	Neal Creek, 3
*p*,*p'*-DDT	--	--	--	--	1.1	0.001	1.1	0.001	--	--	0.000326	Neal Creek, 3
Dieldrin	--	--	--	--	0.24	0.056	0.24	0.056	--	--	0.00031	Neal Creek, 3

Concentrations in micrograms per liter; bold indicates that a detected concentration exceeded the threshold; only detected pesticides with at least one established water-quality threshold are included; benchmark averaging period is the exposure duration appropriate for comparison to the benchmark; --, threshold not established; na, not applicable; WF, West Fork.

Wide-ranging physiological and behavioral changes can occur after exposure to sublethal concentrations of contaminants [68,69,70]. S1 Text summarizes pesticide effects on salmonids and lethal and sublethal effects thresholds for the detected compounds. Endosulfan is highly toxic to fish [71] and has been shown to negatively impact reproduction and olfaction in fish [69,72]. TWA concentrations of endosulfan in Neal Creek exceeded those that caused sublethal stress responses in the freshwater fish *Clarias gariepinus* (2.5–10 ng/L) [73]. The TWA concentrations of other detected compounds did not exceed concentrations from the literature that caused sublethal effects in fish or their prey.

In Mar.–Apr. 2012, endosulfan was detected in grab samples from Neal Creek (73.6 ng/L, endosulfan-I+II) and its tributary, Lenz Creek (82.2 ng/L), at concentrations exceeding the TWA concentrations from the passive samplers and the acute aquatic life benchmark for fish of 50 ng/L [28,32,33]. Chlorpyrifos was detected in Apr. 2012 in Odell Creek (31.4 ng/L), at 63% of the acute benchmark for invertebrates of 50 ng/L and malathion was detected in June 2012 at 135 ng/L, 46% of the acute benchmark for invertebrates of 295 ng/L [28, 33], so these compounds can still pose risks to organisms in the basin. Carbaryl, which can potentiate effects of OP insecticides on salmonids, was detected in grab samples from Neal Creek in 2011 [32], but detected concentrations (27.4 and 8 ng/L) were much less than the acute criterion of 850 ng/L [28].

### Mixtures

Pesticide mixtures are common in the aquatic environment [34,50,74–78], including in salmonid-bearing streams of the Pacific Northwest [79–82]. Passive sampling data cannot elucidate which contaminants co-occur in a stream at any time during the sampler deployment. However, previous discrete sampling from the Hood River basin and broader studies indicate that multiple contaminants were likely simultaneously present in the sampled streams, especially Neal Creek [11,76]. By assuming that all detected contaminants were present at once, we get a worst-case scenario for potential exposure to mixtures for aquatic organisms of interest. The range of detected compounds per passive sampler deployment was 24–36 for Neal Creek and 6–20 for the other sites. A concentration-addition model is broadly applicable for pesticide mixtures, particularly for pesticides with a shared mode of action, and the approach has a low likelihood of underestimating the potential effects of pesticide mixtures [83]. The instance of synergistic pesticide interactions is relatively rare compared to additive effects and typically involves relatively high concentrations of pesticide classes not detected together in this study [77]. Therefore, considering additive effects of co-occurring pesticides is likely reasonable for this screening-level risk assessment [77,84].

**Fig 5:**
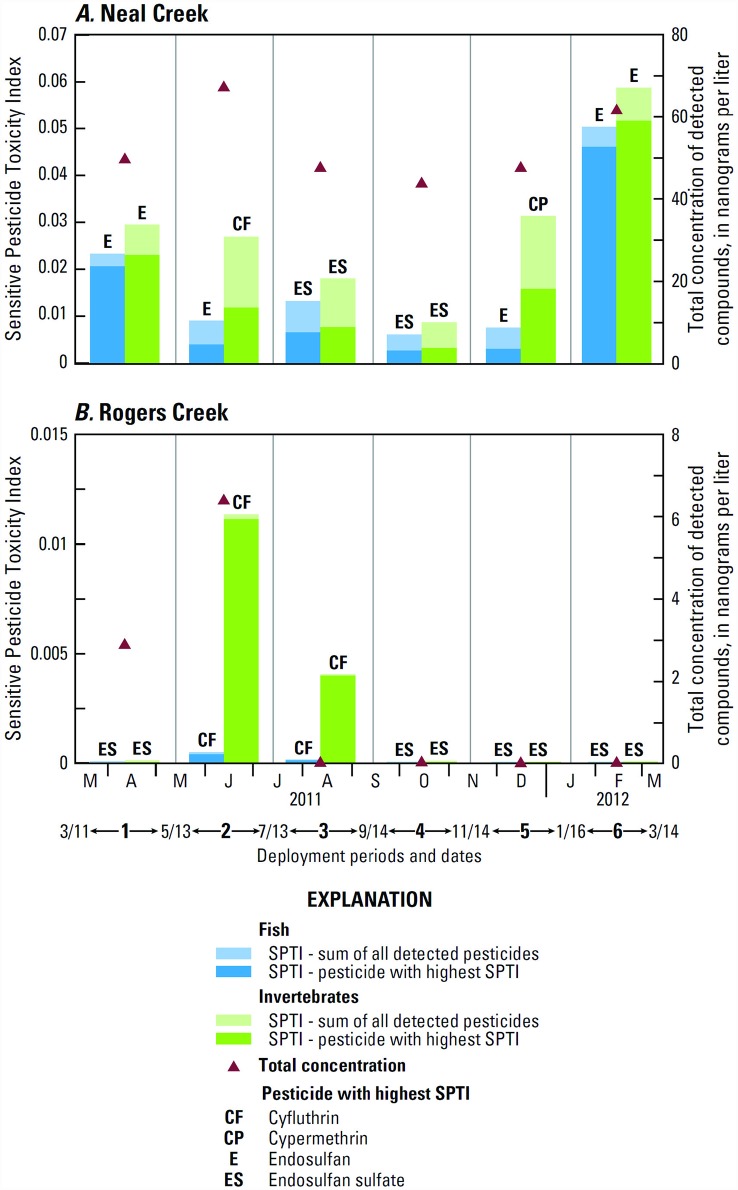
Sensitive Pesticide Toxicity Index values for fish and invertebrates in passive sampler deployments, 2011–12: (A) in Neal Creek; (B) in Rogers Creek. Total concentration is the sum of quantified detected concentrations from the passive samplers. See S4 Table for compound-specific SPTI values and toxicity concentrations used to calculate them.

The Sensitive Pesticide Toxicity Index (SPTI) estimates for Neal and Rogers Creeks indicate that the potential toxicity of the detected mixtures is greater for invertebrates than for fish and greater for Neal Creek than for Rogers Creek (Fig 5). Toxicity indices did not always correlate with total concentration of detected contaminants, since less toxic herbicides are commonly detected at higher concentrations than more toxic insecticides. Even though the detection of cypermethrin in Neal Creek was less than the MQL, its SPTI value was higher than the values for some other detected compounds, because of the extreme toxicity of pyrethroid insecticides to benthic invertebrates. Information on pesticide exposure to aquatic organisms is often incomplete because traditional analytical methods may not be able to detect pesticides at very low, but environmentally relevant, concentrations [85]. None of the SPTIs calculated for passive samples reached the thresholds causing at least 50% toxicity to the water flea *Ceriodaphnia dubia* in 4–8 day tests using data from many published studies (SPTI of 0.1–1) [34]. The detection of carbaryl, a pesticide associated with synergistic toxicity, in grab but not passive samples from Neal Creek in May–July 2011 [32] indicates that potential toxicity to organisms may be underrepresented by the passive sample SPTI.

SPTIs calculated from 2011 ODEQ grab sample data from Neal Creek (collected during passive sampling deployments 1 and 2) were lower than those from passive sampling data (Tables D and F in S4 Table), since the largest contributors to the passive sample SPTIs, endosulfan and cyfluthrin, were not detected in the grab samples. The 3/23/2011 sample from Lenz Creek and the 4/10/2012 sample from Odell Creek had by far the highest total SPTIs among the assessed samples (0.414 and 0.449, respectively, for invertebrates), due to the detections of chlorpyrifos (29 and 31.4 ng/L, respectively). Grab samples collected in late Mar. and early Apr. 2012 (2–3 weeks after the passive sampling ended) had the highest SPTIs for fish (0.193 and 0.207) and among the highest for benthic invertebrates (0.221 and 0.242) due to the detection of endosulfan (48.9 ng/L in Neal Creek and 42 ng/L in Lenz Creek). These higher SPTI values are consistent in general timing with the peak SPTI values from passive sampling Deployment 6, all of which were also attributed to peak endosulfan concentrations. The SPTI for invertebrates from a June 2012 grab sample from Odell Creek was also relatively high (0.189) due to the detection of malathion (135 ng/L).

Synergistic (greater than additive) toxicity to salmonids from OP insecticides such as chlorpyrifos, malathion, and diazinon co-occurring with other OP insecticides or with carbamate insecticides has been well documented [77,79], even at what are normally considered relatively low environmental concentrations (~1,000 ng/L) [80], although they are still much higher than those detected in this study. Sublethal effects can persist long beyond the exposure period, particularly for OPs, and repeated exposures could have increasingly deleterious effects [80,86]. Triazine herbicides such as simazine and hexazinone individually have low toxicity to aquatic organisms, but can potentiate the toxicity of OP insecticides on aquatic invertebrates at concentrations much higher than were detected in this study (25,000–200,000 ng/L [74,75]; maximum grab sample concentrations since 1999 in Neal Creek were 780 [simazine, June 2003] and 95 ng/L [hexazinone, Apr. 2009] [32,33]). The few studies of pesticide mixtures using environmentally relevant concentrations of pesticides commonly detected in Pacific Northwest streams have observed mixed impacts to salmonids. Improvements in low-concentration sampling techniques may allow better understanding of potential threats of low-concentration pesticide mixtures to organisms in the future.

### Fish distribution and potential impacts

**Fig 6:**
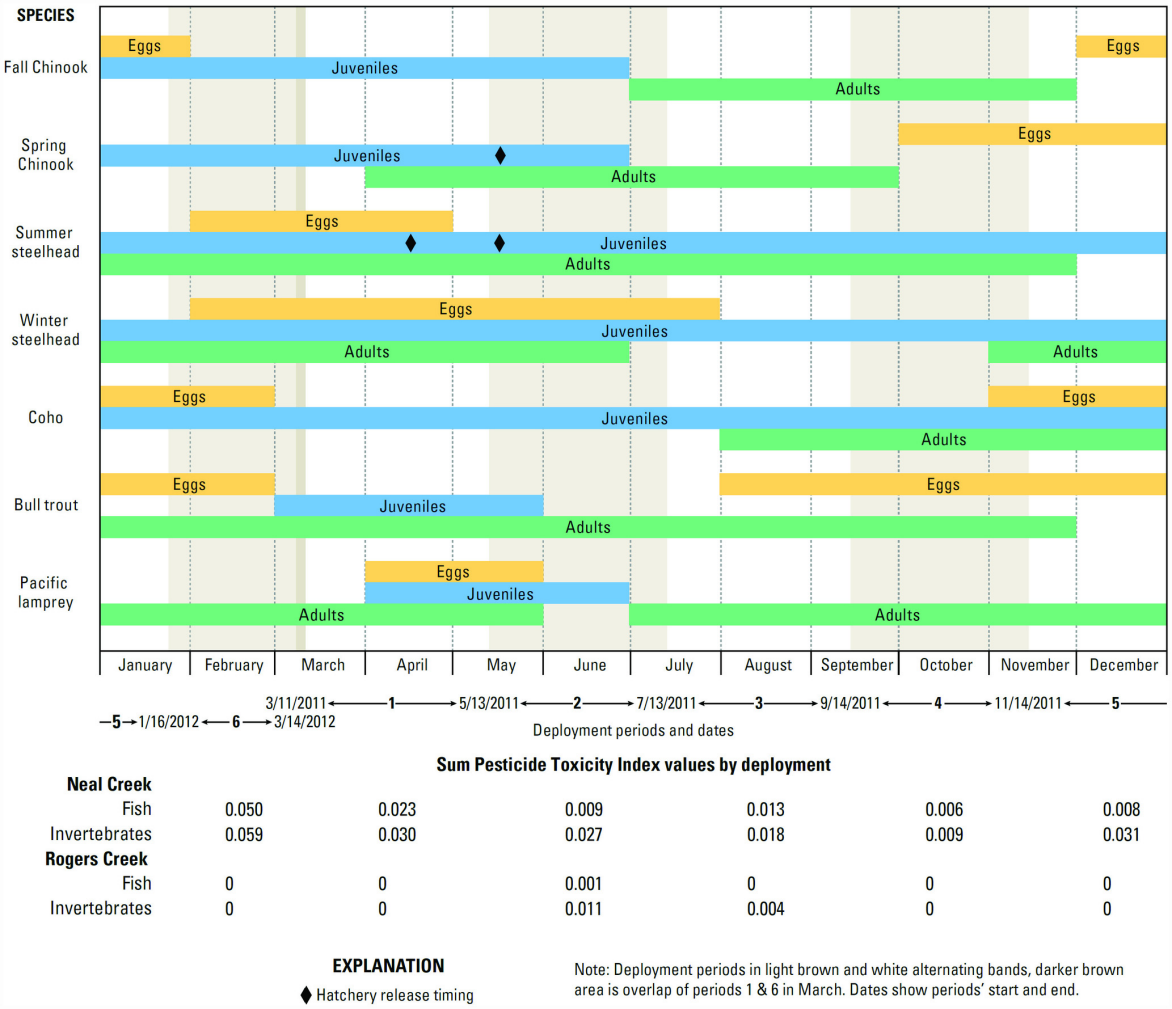
Typical presence of sensitive fish species and life stages in the Hood River basin, Oregon, by month. Sensitive Pesticide Toxicity Indices from each deployment in Neal and Rogers Creeks are shown at the bottom. ([3, 9, 19, 87–90], Jason Seals, Oregon Department of Fish and Wildlife, personal communication, 2015]).

Summer steelhead, winter steelhead, and coho inhabit Neal Creek, while winter steelhead inhabit Rogers Creek [7]. Spring Chinook smolts are raised at Parkdale Fish Facility on Rogers Creek for release into the Middle and West Forks of Hood River [18]. Adults and early life stages of those and other sensitive species are present in the basin [3,9,19,87–90] during the months of peak potential pesticide toxicity in Neal and Rogers Creeks (Fig 6). Contaminant threats can be of particular concern during seasonal peaks in pesticide use (spring and fall) and coincident smolt outmigration and adult return migration of many species [91]. Pesticide presence can be especially of concern for stream-type Chinook, coho, and summer steelhead, which remain in freshwater for months to years before migrating to the ocean [68]. Stream-type spring Chinook collected from the lower Columbia River had low PCB concentrations, but relatively high DDT concentrations [39], similar to environmental patterns revealed by the passive samplers from the Hood River basin sites and similar to body burdens of those contaminants in juvenile Pacific lamprey from the Hood River basin [52]. Concentrations of contaminants from the passive samplers do not appear on their own to be at levels expected to harm salmonids, but cumulative impacts are more difficult to assess [92].

### Potential impacts to macroinvertebrate prey

Besides causing direct health impacts, pesticides can also impair salmonids by limiting the availability of aquatic invertebrate prey [86]. Macroinvertebrate prey are often limited in salmonid-bearing streams, which can result in reduced salmonid growth [68]. Growth is an especially important sublethal endpoint for juvenile salmonids because body size ([68] and references therein, [93]) and lipid content [94–96] are strongly correlated to survival during migration to the ocean and during the first year in the ocean. A previous modeling study [86] showed that highly toxic insecticides (e.g., carbamates, OPs, and pyrethroids) could have short-term impacts to prey communities and subsequent long-term deleterious impacts on salmonid populations. Therefore, management efforts that reduce the presence of those compounds, such as controlling erosion of contaminated riparian soils and minimizing spray drift and runoff could be effective in supporting salmonid growth.

### Other potential stressors

Other chemical and physical stressors to salmonids can also increase pesticide toxicity and the likelihood of sublethal changes. For example, elevated temperatures increased chlorpyrifos and malathion toxicity and disease susceptibility in salmonids [97, 98] and pyrimethanil toxicity to aquatic invertebrates [99]. Neal Creek has been on Oregon’s Category 4(a) listing for high water temperature in the summer since 2002 and is on Oregon’s 2012 year-round 303(d) listings for multiple metals and pesticides [100]. Other streams in the basin are 303(d) listed for sedimentation, OP insecticides, and metals, including copper. Conditions during July–Sept. (when DDT and endosulfan concentrations were among their highest) could be additionally stressful to salmonids due to higher temperatures during lower streamflows. Controlled studies would be necessary to assess any combined effects of chemical and physical stressors on salmonids in this system.

### Compounds of concern

Except for endosulfan, the concentrations of most compounds detected in passive samplers were less than those expected to cause direct toxicity or sublethal effects to salmonids. However, their potential effects on macroinvertebrate prey populations or longer-term salmonid fitness are unknown. Endosulfan concentrations in Neal Creek exceeded the chronic water-quality benchmark for invertebrates and exceeded concentrations inducing stress responses in a freshwater fish. Among insecticides, endosulfans were detected at the highest concentrations in every passive sampling deployment in Neal Creek. They were the largest contributors to total SPTI estimates for passive samples, except those for invertebrates (both sites) and fish (Rogers Creek) when pyrethroids were present. The detection pattern of endosulfans was similar to that of DDTs, except during the deployment when endosulfan use was expected and the concentrations peaked. DDTs and dieldrin appear to also be of concern, as their concentrations in Neal Creek exceeded U.S. EPA water-quality criteria for human health and the TWA concentration of DDT was close to the chronic water-quality criterion for freshwater organisms [29]. TWA concentrations of pyrethroid insecticides were not at concentrations that caused toxicity or sublethal effects in published studies, but their extreme toxicity and their potential to cause long-term changes to macroinvertebrate communities, even with exposures of only a few hours [70] indicate that pyrethroids may be of concern at shorter time scales. Although infrequently, endosulfan, chlorpyrifos, and malathion were present in ODEQ grab samples at concentrations near or exceeding acute water-quality benchmarks.

### Impacts of watershed management activities on in-stream pesticides

Watershed management actions in the basin enacted to restore streamflows, improve fish habitat, or reduce pesticide inputs to streams can affect the potential for in-stream pesticide exposures on fish species of concern. Fish passage barrier removals can increase the number and extent of fish species of concern [10], as has been demonstrated for Pacific lamprey in the basin [101]. Efforts to reduce pesticide transport to streams, such as canal infrastructure modifications [102] and riparian vegetation planting [10] in concert with efforts to reduce reliance on insecticides, such as the implementation of Best Management Practices and innovative Integrated Pest Management activities by fruit growers [103], were implemented in the basin throughout the early 2000s.

Many of the pesticides that were detected frequently or at concentrations exceeding water-quality benchmarks through the 2000s, such as azinphos methyl, malathion, and phosmet, were not detected in passive samplers deployed in Mar. 2011–2012. Chlorpyrifos was the only OP insecticide detected in the passive samplers. It was detected at TWA concentrations of 0.2 ng/L or less, much less than previous grab sample concentrations (30–480 ng/L) [32], but was present during all six deployments at Neal Creek [33]. CUPs were present in streams throughout the year, but peak concentrations coincided with peak timing of use (herbicides in spring; insecticides in late winter to summer). Concentrations of pyrimethanil, the only CU fungicide detected in passive samplers, peaked during and after its peak season of use (fall), when it is used in fruit packing facilities against postharvest decay, [46]. Passive sample SPTI estimates were highest during Jan.–Mar. 2012 (for Neal Creek) and May–July 2011 (for Rogers Creek), as were total insecticide concentrations.

Some classes of CU insecticides pose extreme risks to sensitive fish species and their macroinvertebrate prey, partly due to their potential for synergistic toxicity (OPs and carbamates) or direct toxicity (pyrethroids). The presence in Pacific Northwest orchards of the spotted wing drosophila (*Drosophila suzukii*), an invasive fruit fly that can devastate crops by attacking ripening small fruits such as cherries, has necessitated increased use of malathion in Oregon in recent years, despite pesticide reduction efforts in many areas [33]. OPs (malathion, diazinon, and dimethoate) and other highly toxic insecticides, such as pyrethroids and neonicotinoids are cited as the most effective treatments against *D*. *suzukii* in the Pacific Northwest [104,105]. Rotations of several pyrethroids, malathion, and spinosad are being used in the mid-Columbia region for long-lasting *D*. *suzukii* control and to minimize the likelihood of resistance to any single compound. Aerially applied malathion is cited as a particularly effective application method for *D*. *suzukii* control [104], but could lead to direct spray or drift into surface waters. Continuing efforts to reduce insecticide drift and runoff during late winter to summer and careful management against *D*. *suzukii* could protect early life stages of several sensitive fish species that are present in the basin. Appropriate assessment of pesticide-related risks to organisms also requires that sampling programs change the suites of analyzed compounds as use patterns change. S5 Table includes a list of pesticides used in the basin’s orchards as of 2015, including compounds that have not been analyzed in recent sampling efforts.

Limited pesticide concentration data from sediments in the basin indicate that concentrations of *p*,*p’*-DDT and its degradate *p*,*p’*-DDE were approximately 10 times higher in Neal Creek in 1998 [32] than in the mainstem Hood River in 2011 [52]. Efforts to minimize erosion of contaminated riparian soils, runoff from forest and developed roads, and sedimentation from irrigation return flows could reduce further inputs of legacy hydrophobic contaminants to streams and bioaccumulation into organisms, as has been reported in larval Pacific lamprey from the mainstem Hood River [52]. Such measures can also reduce in-stream sedimentation, which is one of the five primary limiting factors for anadromous salmonid production in the basin [9]. Finally, reporting updated fish habitat use information following restoration actions can be used to help prioritize streams of interest for future water-quality monitoring and/or for pesticide reduction efforts. Data presented here can be used as updated baseline information with which to evaluate impacts of future watershed management actions.

## Conclusions

Year-round passive sampling described here offers several new insights regarding the presence of pesticides and other contaminants in Hood River basin streams. Previously, no wintertime pesticide and few fall concentration data for the basin were available, so seasonal patterns of detection could not be assessed. Twenty pesticides in Neal Creek and 5 in Rogers Creek were detected during all deployments, indicating that many pesticides were present throughout the year (i.e., during some portion of every 2-month sampling period). Although this does not indicate continuous presence in streams throughout each deployment, it offers useful information for the assessment of potential threats to salmonids in the basin and provides a baseline comparison for future passive sampling data that can help target future sampling efforts and management goals. The presence of pesticides in upper-watershed streams was also previously not well characterized, particularly during the fall, when herbicide application for forestry site preparation is common. These results reveal that CUPs are entering upper-watershed streams.

A key finding from this study is the presence of numerous compounds not previously characterized in the basin’s surface waters, including pesticides that are known to be used in the basin (e.g., pyrethroids) and hydrophobic compounds that are known to persist in the environment and to bioaccumulate (e.g., PBDEs, DDT degradates, endosulfans, chlordanes). Very low thresholds of detection with passive samplers allowed the detection of some compounds at concentrations that would not be detectable using traditional point-in-time grab sampling analyses. For example, the MQLs for DDTs, endosulfans, and dieldrin from ODEQ’s grab samples in Mar.–July 2011 were 23–26 ng/L [32], whereas they were detected at TWA concentrations less than 1 ng/L from the passive samplers in Neal Creek during the same time period. While these low concentrations may not be of concern on their own, they provide context with which to assess the overall status of contaminant stressors to salmonids and their prey, such as identifying pesticide mixtures in the basin’s streams. Another notable finding is that polychlorinated biphenyls (PCBs) were not detected during any deployment in this study.

PBDEs and pesticides were detected year round in passive samplers in Mar. 2011–2012. CU herbicides were detected at among the highest concentrations, but did not contribute to estimated toxicity of pesticide mixtures as much as insecticides. SPTI estimates showed that the highest sum concentrations of all contaminants did not always correlate to the highest estimated toxicity of mixtures; total insecticide concentration was a better indicator of toxicity. Nearly all CUPs were present in streams beyond the seasons when they were expected to have been used (Table 4), especially in Neal Creek. Neal Creek had more compounds detected, higher concentrations, and higher SPTIs for fish and invertebrates than Rogers Creek. However, the detected concentrations and SPTIs for both creeks were generally low compared to thresholds of concern for salmonid species and their prey. SPTIs for 2011–2012 grab samples highlighted that higher short-term concentrations of insecticides resulted in the highest SPTIs. CU herbicides and some legacy pesticides were detected in two upper-watershed sites during the single passive sampler deployment during fall 2011.

At the year-round sampling sites, DDTs were among the most consistently detected legacy insecticides. Concentrations of DDTs were relatively consistent among deployments in Rogers Creek, whereas concentrations in Neal Creek peaked in late spring and summer. Concentrations of endosulfan followed the same pattern as DDTs in Neal Creek, except during the Jan.–Mar. 2012 deployment, when endosulfan was most likely used and the detected concentration was highest. Endosulfan concentrations in Neal Creek exceeded the chronic water-quality benchmark for invertebrates and concentrations that induced stress responses in the freshwater fish *C*. *gariepinus* [73], but seasonally high concentrations would not be expected after the insecticide is fully phased out in 2016.

Data presented here can be used as baseline data with which to evaluate impacts of future watershed management actions. ODEQ began sediment sampling for hydrophobic contaminants in 2014. Future monitoring could also include sampling riparian soils to help prioritize areas for erosion prevention measures, or assessment of groundwater-surface water connectivity to determine whether year-round DDT and endosulfan detections could be due to groundwater inputs to Neal Creek. Continuing efforts to reduce spray drift and runoff to streams, particularly if the use of highly toxic insecticides (e.g., OPs and carbamates) increases, could be effective in continuing to support salmonid growth. Overall, most individual pesticides detected in this study were not measured at concentrations expected to cause direct impacts to salmonids and it appears that efforts to reduce in-stream pesticide presence in the basin are achieving the desired results. However, cumulative toxicity of pesticide mixtures could be of concern over shorter time scales than were measured by passive samplers, particularly if OP and pyrethroid use increases in response to *D*. *suzukii* or other pest infestations. Based on the available data, direct toxicity to salmonids from in-stream pesticide exposure is unlikely, but indirect impacts, such as reduced fitness due to cumulative exposures or negative impacts to invertebrate prey populations, which could have long-term deleterious impacts on salmonid populations in the basin, are not known.

## Supporting Information

S1 Table Compounds sampled with semipermeable membrane devices (SPMDs) and polar organic continuous integrative samplers (POCIS), Hood River basin, Oregon, 2011–2012. (XLSX)

S2 Table Quality assurance data for passive samplers, Hood River basin, 2011–2012. (XLSX)

S3 Table Pesticide grab sample data collected from the Hood River basin, Oregon, by Oregon Department of Environmental Quality, 2011–2012. (XLSX)

S4 Table Sensitive Pesticide Toxicity Index values for passive and grab samples collected from the Hood River basin, Oregon, 2011–2012 and toxicity data used for those calculations. (XLSX)

S5 Table Pesticides used on orchards in the Hood River basin, Oregon, as of 2015. (XLSX)

S1 Text Literature review on pesticide effects on salmonids. (PDF)

S2 Text Discussion of comparisons between passive and discrete sample data. (PDF)
