# Supplementary material for: Year-Round Monitoring of Contaminants in Neal and Rogers Creeks, Hood River Basin, Oregon, 2011-12, and Assessment of Risks to Salmonids
Source: PLoS One. 2016 Jun 27;11(6):e0158175. doi: 10.1371/journal.pone.0158175 (PMC4922572; doi:10.1371/journal.pone.0158175)
Supplement: S1 Text — (PDF) [file pone.0158175.s006.pdf]

S1 Text. Literature review on pesticide effects on salmonids

A review of literature used to assess potential risks to salmonids and their prey from individual compounds and mixtures of contaminants detected in the passive samplers in the Hood River basin in March 2011–12 follows. Data from the available literature indicated that the detected concentrations were largely too low to warrant concern for direct toxicity or sublethal effects, so this summary was excluded from the main body of the text.

Compounds detected in the passive samplers vary widely in their potential threats to salmonid species and their prey. Several have been shown to accumulate rapidly into fish or invertebrate tissues [1]. For example, DDT bioaccumulates and persists in tissues, whereas hexachlorobenzene (HCB) and boscalid are metabolized and do not persist in tissues once the exposure source is eliminated [1-3]. Many of the detected compounds (including 2,4-D, chlordane, DDTs, dieldrin, endosulfan, HCB, and simazine) are endocrine disruptors [4] or cause other adverse sublethal effects such as impaired mobility, altered stress response, or reduced growth [5-8]. Moreover, some compounds cause irreversible impacts (e.g., inhibition of acetylcholinesterase enzymes by organophosphate [OP] insecticides), whereas other effects may be reversible (e.g., inhibition of acetylcholinesterase enzymes by carbamate insecticides) [9].

Lethal and sublethal effects thresholds for detected currently used pesticides

2,4-D is slightly to moderately toxic to salmonids and aquatic invertebrates [10]. The 50% lethal concentration (LC50) for Chinook salmon (*Oncorhynchus tshawytscha*), the most sensitive salmonid species for which there are data, is 4,800,000 ng/L [10]. Oruc et al. [11] reported that sublethal concentrations of 2,4-D (87,000,000 ng/L) alone and in combination with the OP insecticide azinphos-methyl caused oxidative stress in freshwater fish. The maximum time-weighted average (TWA) concentration in this study was 250 ng/L.

Chlorsulfuron is practically nontoxic to freshwater fish and invertebrates [12]. No Observed Effect Concentrations (NOECs) are 32,000,000 ng/L for rainbow trout (*O. mykiss*) and 20,000 ng/L for the water flea *Daphnia magna* with chronic exposures [12]. The maximum TWA concentration in this study was 27 ng/L.

Hexazinone is highly mobile and widely used throughout the U.S. and is practically to slightly toxic to aquatic invertebrates and fish [13]. LC50s are in the range of 246,000,000-317,000,000 ng/L for juvenile coho salmon (*O. kisutch*) and greater than 180,000,000 ng/L for rainbow trout [5,13,14]. Exposure to hexazinone in water at concentrations of 100,000 ng/L decreased gill sodium- and potassium-ATPase activity, a marker for development of seawater tolerance, in Atlantic salmon (*Salmo salar*) smolts, but did not affect growth, brain cholinesterase activity, or plasma glucose or cortisol levels [15]. Chronic exposures to hexazinone (21 and 39 days) adversely affected reproduction and survival of the water flea *D.magna* at concentrations of 50,000 and 81,000 ng/L, respectively, and growth of fathead minnows (*Pimephales promelas*) at 35,500 ng/L [5]. The maximum TWA concentration in this study was 15 ng/L.

Metsulfuron methyl has very low toxicity to aquatic organisms (LC50 > 150,000,000 ng/L for rainbow trout and *D. magna*) [16]. Exposures of 100,000,000 ng/L caused lethargy and erratic swimming in rainbow trout, but no effects were demonstrated in chronic exposures of 4,500,000 ng/L [16]. The maximum TWA concentration in this study was 70 ng/L.

Simazine is practically nontoxic to slightly toxic to rainbow trout and practically nontoxic to moderately toxic to aquatic invertebrates in acute exposures, but is highly toxic to aquatic vascular plants [17]. Potential chronic effects to freshwater fish and invertebrates are not known [18]. Simazine concentrations of 2,000 ng/L impacted olfaction in Atlantic salmon [19]. It is a hormone disruptor in humans [4,18,20] and was banned in Europe for its high potential to contaminate groundwater [21]. The maximum TWA concentration in this study was 36 ng/L.

Pyrethroid insecticides are of great concern due to their extreme toxicity to fish and invertebrates, with LC50 values in the range of 2–140 ng/L in water and 4–110 ng/g in sediment for aquatic invertebrates and 600–19,000 ng/L for fish [22-24]. They have been shown to bioaccumulate in wild freshwater fish [24]. Invertebrates exposed to pyrethroids exhibit a variety of behavioral effects that have the potential to increase their vulnerability to predators, including changes in feeding rates, paralysis and loss of coordination, hyperactivity, and abandonment of protective cases by case-building

caddisflies (order: Trichoptera) [23]. Cyfluthrin, the only pyrethroid insecticide detected at quantified concentrations, is among the most toxic of the pyrethroids. It has been shown to reduce aquatic invertebrate growth at sediment concentrations of 0.46–0.77 ug/g organic carbon [23], cause a variety of biochemical and physiological changes to carp (*Cyprinus carpio* L.) fingerlings with water exposures of 10,000 ng/L for 2 and 7 days [25], and impair the ability of fathead minnows to resist extreme temperatures with water exposures of 170–1,110 ng/L [26]. Cyfluthrin exposures of 18 ng/L reduced growth and elicited behavioral effects on early life stages of rainbow trout [27]. The maximum TWA concentration in this study was 0.140 ng/L.

Organophosphate (OP) pesticides disrupt a variety of olfactory-mediated behaviors in salmonids, including homing, migration, predator avoidance [19,28,29] and can have synergistic toxicity when present in combination with carbamate or other OP pesticides [30-32]. Due to its high toxicity to threatened and endangered Pacific salmonids and their invertebrate prey, the use of the OP insecticide chlorpyrifos was recently restricted near salmonid-bearing streams [33]. Acute toxicity of chlorpyrifos is exacerbated by increased water temperatures, compounding potential threats to sensitive salmonid species [33]. Sublethal concentrations can impair olfactory-mediated behaviors of juvenile coho salmon at environmentally realistic concentrations (625—2,500 ng/L) [34,31]. Chlorpyrifos exposures of 120—2,680 ng/L caused reproductive effects and reduced growth in fathead minnows [33]. Environmental concentrations of 2,300 ng/L chlorpyrifos reduced the abundance of macroinvertebrate prey items for salmonids [31,35]. The maximum TWA concentration of chlorpyrifos in this study was 0.200 ng/L.

Pyrimethanil is slightly toxic to fish and moderately toxic to invertebrates [3]. Reported LC50 concentrations for fish are 10,100,000–10,600,000 ng/L [3,6]. Sublethal effects to fish are not known. Reported LC50 values for aquatic invertebrates are 3,000,000–8,000,000 ng/L, but deleterious impacts to reproduction, mobility, and emergence are in the range of 1,000,000–8,000,000 ng/L [3,6]. Additionally, toxicity of pyrimethanil to aquatic invertebrates increases at higher temperatures [6]. The maximum TWA concentration in this study was 15 ng/L.

Toxicity and sublethal effects of the herbicide degradates 3,4-DCA and 3,5-DCA are less well studied than those of many pesticide parent products. However, toxicity studies indicate that LC50 concentrations for benthic invertebrates and zebrafish (*Danio rerio*) were 2,500,000–8,500,000 ng/L and 57,000,000–62,000,000 ng/L for zooplankton species [36,37]. 3,5-DCA appears to be less toxic than its parent compounds to fish and aquatic invertebrates [38]. Zebrafish showed no effects to survival, hatching, or growth after a 28-day exposure to 3,5-DCA at 1,000,000 ng/L [38]. The U.S. EPA has classified 3,5-DCA as moderately toxic to aquatic organisms based on acute exposures. However, a parent compound, iprodione, is moderately toxic to freshwater fish and highly toxic to aquatic invertebrates with acute exposures [38]. The maximum TWA concentrations in this study were 190 ng/L for 3,4-DCA and 220 ng/L for 3,5-DCA.

Triclopyr is available in two formulations. The butoxyethyl ester (BEE) formation is highly toxic to salmonids, whereas the triethylamine salt (TEA) formation is practically nontoxic to salmonids and other fish species [39,42]. Both formulations are potentially used in the Hood River basin for forestry or rights-of-way weed control. The BEE formulation is highly toxic to salmonids, with LC50 concentrations in the range of 740,000–2,700,000 ng/L, and expected increases in toxicity with higher temperatures [39]. Kreutzweiser et al. [39] determined that the toxicity of triclopyr to rainbow trout and Chinook salmon increased substantially during the first six hours of exposure, with less rapid increases in toxicity for exposures up to 24 hours. They also reported that even when mortality did not occur for up to 24 hours, disorientation, surfacing, and gill flaring were observed frequently within the first hour of triclopyr BEE exposure and most fish displaying behavioral changes did not recover [39]. Triclopyr BEE concentrations of 700–800 ng/L sustained for 24 hours were shown to cause substantial mortality to salmonids in a laboratory setting [39]. However, it appears to typically degrade rapidly in surface water after applications to Pacific Northwest forests and pastures [39-41]. Triclopyr is transported to streams through overland runoff following precipitation events, but water exposure from overhead spray appears to pose a greater risk to salmonids [39,41]. Aquatic insects appear to be less sensitive to triclopyr formulations than are salmonids [39,42]. The maximum TWA concentration of triclopyr in this study was 250 ng/L.

Boscalid is persistent, moderately toxic to fish, and moderately to highly toxic to aquatic invertebrates [3,43]. It caused lethargy, narcosis, and extended yolk sacs in rainbow trout at concentrations of 241,000 ng/L, reduced fecundity in water fleas (*Daphnia spp.*) at 1,540,000 ng/L, and reduced emergence in midges (*Chironomus spp.*) at 4,000,000 ng/L [3,43,44]. It is expected to accumulate moderately in fish, but not to persist in tissues once exposure ceases [3,43]. Bioaccumulation

in benthic invertebrates has not been studied, but is expected to be important because boscalid is strongly sediment-bound [3,43]. The maximum TWA concentration of boscalid in this study was 180 ng/L.

Lethal and sublethal effects thresholds for detected legacy compounds

Several legacy compounds detected in the passive samplers cause acute toxicity or sublethal effects to salmonids or their invertebrate prey, even at low exposure concentrations. Organochlorine (OC) pesticides, including DDT and its breakdown products, DDD and DDE, can cause immune suppression, physical and developmental defects, reduced growth, and reproductive changes, such as disruption of sperm production and changes to the sex ratio of offspring [4]. DDT rapidly accumulated in aquatic invertebrates to several thousand times of exposure levels with exposure concentrations as low as 80 ng/L [1]. Dietary exposure is an important pathway for uptake into fish [1]. DDE altered the thyroid hormone feedback system and the steroid-metabolizing enzyme in the liver and other organs in Atlantic salmon parr with five-day water exposures of 10,000 ng/L [45]. The maximum TWA concentration in this study was 1.790 ng/L for total DDTs.

Chlordane is very highly toxic to salmonids and aquatic invertebrates, with LC50s of 800–4,700 ng/L for rainbow trout and 1,500 ng/L for the stonefly *P. californica* in 96-hour exposures [46]. Adverse sublethal effects were observed in brook trout (*Salvelinus fontinalis*) with chronic exposures of 320 ng/L [46]. Reduced hatches were observed after continuous exposures of 800 ng/L, and concentrations of 1,700 ng/L resulted in some deaths of second-generation sheepshead minnows (*Cyprinodon variegatus*) [46]. The maximum TWA concentration in this study was 0.008 ng/L for total chlordanes.

Dieldrin is highly toxic to fish and aquatic invertebrates. For many salmonid species, LC50s are in the range of 6,000-12,000 ng/L, whereas they are lower for bluegill (*Lepomis macrochirus*) (3,100 ng/L) and aquatic invertebrates (500–7,600 ng/L) [1,10,47]. The maximum TWA concentration of dieldrin in this study was 0.310 ng/L.

Hexachlorobenzene has been shown to assimilate rapidly and to bioconcentrate in multiple species of aquatic invertebrates and fish through water and dietary exposures of contaminated sediment and algae, but it is quickly removed from tissues after the exposure source is removed [1,2,48]. The LC50 is greater than 50,000 ng/L for coho salmon, but 12,000 ng/L for largemouth bass (*Micropterus salmoides*) and bluegill [1]. The maximum TWA concentration in this study was 0.015 ng/L.

Water-quality criteria have not been established for PBDEs, which have been shown to accumulate in salmonids [49], increase the susceptibility of juvenile Chinook salmon to diseases following environmentally relevant dietary exposure [50], and are known to cause endocrine disruption and neurotoxicity [51,52]. Chou et al. [53] reported decreases in distance moved and percent of time active, important indicators for predator avoidance and migration behaviors, in juvenile zebrafish with dietary exposures to PBDE-47 at environmentally relevant doses (leading to tissue residues of 11, 189, and 1924 ng/g). Similar neurobehavioral impairments, along with poor predation performance, were observed in an estuarine minnow (*Fundulus heteroclitus*) following post-fertilization exposures to PBDE-71 at concentrations of 1 and 10 ng/L [53]. Chinook salmon from the Pacific coasts of Oregon and British Columbia have significantly higher PBDE loads compared to other wild salmonids around the world [54]. In their study, Dietrich et al. [54] found that juvenile Chinook salmon accumulated many of the same PBDE congeners as were detected in this study (PBDE-28, -47, -99, -100, -153, and -154) through dietary exposure at a wide range of contaminant concentrations in food (0.7 to 1500 ng PBDEs per gram of food), encompassing the range of concentrations examined in similar studies with Atlantic salmon, found in hatchery foods, and in the stomach contents of wild Chinook. Except for PBDE-49, which was shown to increase due to debromination of PBDE-99 to PBDE-49, they found that PBDE assimilation increased with higher lipid levels of food sources. The maximum TWA concentration in this study was 0.489 ng/L for total PBDEs.

Pesticide mixtures in the Pacific Northwest

Tierney et al. [29] observed that environmentally relevant and higher concentrations of a mixture of 10 pesticides detected frequently in a British Columbia river reduced olfactory sensory performance in rainbow trout and inhibited a detoxifying response that occurred following lower-dose exposures. Their mixture contained simazine, chlorpyrifos, endosulfan, and 7

pesticides not detected in this study, mostly at concentrations exceeding those detected in this study. King et al. [55,56] found no significant effect on a host of health and reproductive biomarkers in coho salmon juveniles exposed to a mixture of 12 pesticides commonly detected in western Washington streams. They observed differences in adult return rates among treatment groups, including a 40% reduction in the number of returning adults one year following exposure from fertilization through smoltification, although overall patterns in return rates were not consistent among different years' cohorts [56]. The pesticide mixture used in those studies included some compounds detected in this study (2,4-D [690 ng/L], simazine [420 ng/L], triclopyr [740 ng/L], and pentachlorophenol [120 ng/L] plus eight other pesticides or degradates [60–590 ng/L]), but all concentrations were higher than the TWA concentrations presented in this study.

Fish distribution and potential impacts

During some high-energy use life stages, including reproduction and smolting, salmonids can be exposed to elevated risks of contaminant effects as metabolic changes redistribute contaminants within the body [57]. Early life stage contaminant exposure via external sources or maternal transfer can also lead to long-term impairments of immune function, growth, stress response, osmoregulation, and marine survival [58]. Ross et al. [58] noted that reduced fitness and increased vulnerability to various stressors are more insidious threats to salmonids than direct toxicity from pesticide exposure. Modeled short-term exposures of environmentally realistic (although higher than concentrations detected in the Hood River basin) OP and carbamate insecticide concentrations reduced growth rates and size at ocean entry for juvenile Chinook with resultant reductions in spawner abundance in the affected population over 20 years [59]. Likewise, the modeled population abundance decreased as a result of various sublethal impacts of contaminant exposure, most notably reduction of first-year survival [60].

Potential impacts to invertebrate prey

In modeled simulations, carbamates were more likely to reduce long-term salmonid population growth due to reduced prey availability, as opposed to OPs, which reduced population growth by altering feeding behavior [35]. Short-term exposures also reduced long-term growth rates, with larger reductions following multiple brief exposures [35]. This pattern was attributed to the longer recovery time for the macroinvertebrate prey community following multiple exposures [35], consistent with findings that repeated seasonal pesticide pulses reduced the base population size of a macroinvertebrate community [61].

References

1. Johnson WW, Finley MT. Handbook of acute toxicity of chemicals to fish and aquatic invertebrates: Summaries of toxicity tests conducted at Columbia National Fisheries Research Laboratory, 1965-78. Washington, D.C.: U.S. Fish and Wildlife Service; 1980. Resource Publication No. 137.

2. Nebeker AV, Griffis WL, Wise CM, Hopkins E, Barbitta JA. Survival, reproduction and bioconcentration in invertebrates and fish exposed to HCB. Environ Toxicol Chem. 1989;8:601-11.

3. Elskus AA. Toxicity, sublethal effects, and potential modes of action of select fungicides in freshwater fish and invertebrates: U.S. Geological Survey; 2012. Open-File Report No.: 2012-1213. http://pubs.usgs.gov/of/2012/1213/pdf/ofr2012-1213.pdf

4. Mnif W, Hassine AIH, Bouaziz A, Bartegi A, Thomas O, Roig B. Effect of endocrine disruptor pesticides – a review. Int J Environ Res Public Health. 2011;8:2265-303. http://www.mdpi.com/1660-4601/8/6/2265/htm

5. Leyhe J. Hexazinone analysis of risks to endangered and threatened salmon and steelhead. U.S. Environmental Protection Agency, Office of Pesticide Programs, Environmental Field Branch; 2004 [cited 2015 Sep 11]. Available from: http://www.epa.gov/espp/litstatus/effects/hexazin-analysis.pdf

6. Seeland A, Oehlmann J, Müller R. Aquatic ecotoxicity of the fungicide pyrimethanil: Effect profile under optimal and thermal stress conditions. Environ Pollut. 2012;168:161-9.

7. Panger M, Hetrick J. Risks of cyfluthrin and *beta*-cyfluthrin use to federally threatened bay checkerspot butterfly (*Euphydryas editha bayensis*), valley elderberry longhorn beetle (*Desmocerus californicus dimorphus*), California tiger salamander (*Ambystoma californiense*), central California dstinct population segment, and delta smelt (*Hypomesus transpacificus*), and the federally endangered California clapper rail (*Rallus longirostris obsoletus*), California freshwater shrimp (*Syncaris pacificus*), California tiger salamander (Ambystoma californiense) Sonoma County distinct population segment and Santa Barbara County distinct population

segment, San Francisco garter snake (*Thamnophis sirtalis tetrataenia*), and tidewater goby (*Eucyclogobius newberryi*) [Internet]. U.S. Environmental Protection Agency, Office of Pesticide Programs, Environmental Fate and Effects Division; 2013 [cited 2015 Sep 11]. Available from: http://citeseerx.ist.psu.edu/viewdoc/download?doi=10.1.1.299.9688&rep=rep1&type=pdf

8. Cheng Y, Ekker M, Chan, HM. Relative developmental toxicities of pentachloranisole and pentachlorophenol in a zebrafish model (*Danio rerio*). Ecotoxicol Environ Saf. 2015;112:7-14.
9. Laetz CA, Baldwin DH, Hebert VR, Stark JD, Scholz NL. Elevated temperatures increase the toxicity of pesticide mixtures to juvenile coho salmon. Aquat Toxicol. 2014;146:38-44.
10. Munn MM, Gilliom RJ. Pesticide toxicity index for freshwater aquatic organisms. Sacramento (CA): U.S. Geological Survey, National Water-Quality Assessment Program; 2001. Scientific Investigations Report No. 01-4077.
11. Oruc EO, Sevgiler Y, Uner N. Tissue-specific oxidative stress responses in fish exposed to 2,4-D and azinphosmethyl. Comp Biochem Physiol Pharmacol Toxicol Endocrinol. 2004;137:43-51.
12. Reregistration Eligibility Decision (RED) for Chlorsulfuron [Internet]. U.S. Environmental Protection Agency; 2005 [cited 2015 Sep 11]. Available from: http://archive.epa.gov/pesticides/reregistration/web/pdf/chlorsulfuron_red.pdf
13. Reregistration Eligibility Decision (RED) for Hexazinone. U.S. Environmental Protection Agency; 1994 [cited 2015 Sep 11]. Available from: http://archive.epa.gov/pesticides/reregistration/web/pdf/0266.pdf
14. Wan MT, Watts RG, Moul DJ. Evaluation of the acute toxicity to juvenile Pacific salmonids of hexazinone and its formulated products: Pronone 10G, Velpar® L, and their carriers. Bull Environ Contam Toxicol. 1988;41:609-16.
15. Nieves-Puigdoller K, Björnsson BT, McCormick SD. Effects of hexazinone and atrazine on the physiology and endocrinology of smolt development in Atlantic salmon. Aquat Toxicol. 2007;84:27-37.
16. Pesticide information profile for metsulfuron-methyl [Internet]. Corvallis (OR): Extension Toxicology Network; c. 1996 [updated 1996; cited 2015 Sep 10]. Available from: http://extoxnet.orst.edu/pips/metsulfu.htm
17. Turner L. Simazine analysis of risks to endangered and threatened salmon and steelhead [Internet]. Washington, DC: U.S. Environmental Protection Agency, Office of Pesticide Programs Environmental Field Branch, 2003 Jul. [cited 2015 Sep 1]. Available from: http://www.epa.gov/espp/litstatus/effects/simazine-final.pdf
18. Reregistration Eligibility Decision (RED) for Simazine [Internet]. U.S. Environmental Protection Agency; U.S. Environmental Protection Agency; 2006 [cited 2015 Sep 11]. Available from: http://archive.epa.gov/pesticides/reregistration/web/pdf/simazine_red.pdf
19. Tierney KB, Baldwin DH, Hara TJ, Ross PS, Scholz NL, Kennedy, CJ. Olfactory toxicity in fishes. Aquat Toxicol. 2010;96:2-26.
20. McKinlay R, Plant JA, Bell JNB, Voulvoulis N. Endocrine disrupting pesticides: Implications for risk assessment. Environ Int. 2008;34:168-83.
21. Commission of the European Union. Commission decision as of 10 March 2004 concerning the non-inclusion of simazine in Annex I to the Council Directive 91/414/EEC and the withdrawal of authorisations for plant protection products containing this active substance. The Official Journal of the European Union; 2004 [cited 2015 Nov 25]. Document no.: 32004D0247. Available from: http://eur-lex.europa.eu/legal-content/EN/TXT/PDF/?uri=CELEX:32004D0247&from=EN
22. Luo Y, Zhang M. Environmental modeling and exposure assessment of sediment-associated pyrethroids in an agricultural watershed. PLoS ONE. 2011;6(1):1-10. http://www.ncbi.nlm.nih.gov/pmc/articles/PMC3016336/pdf/pone.0015794.pdf
23. Palmquist K, Salatas J, Fairbrother A. Pyrethroid insecticides – use, environmental fate, and ecotoxicology, in Farzana P, editor. Insecticides – Advances in Integrated Pest Management: InTech Publishing; 2012. http://cdn.intechopen.com/pdfs-wm/25677.pdf
24. Corcellas C, Eljarrat E, Barceló D. First report of pyrethroid bioaccumulation in wild river fish: a case study in Iberian river basins (Spain). Environ Int. 2015;75:110-6.
25. Sepici-Dinçel A, Benli ACK, Selvi M, Sarikaya R, Şahin D, Özkul IA, et al. Sublethal cyfluthrin toxicity to carp (*Cyprinus carpio* L.) fingerlings: Biochemical, hematological, histopathological alterations. Ecotoxicol Environ Saf. 2009;72:1433-9.
26. Heath S, Bennett WA, Kennedy J, Beitinger TL. Heat and cold tolerance of the fathead minnow, *Pimephales promelas*, exposed to the synthetic pyrethroid cyfluthrin. Can J Fish Aquat Sci. 1994;51:437-40.
27. EDSP weight of evidence analysis of potential interaction with estrogen, androgen, or thyroid pathways – chemical: cyfluthrin. Final Report. Washington, DC: U.S. Environmental Protection Agency, Office of Pesticide Programs, Office of Science Coordination and Policy; 2015. http://www.epa.gov/sites/production/files/2015-06/documents/cyfluthrin-128831_2015-06-29_txr0057163.pdf
28. Sandahl JF, Baldwin DH, Jenkins JJ, Scholz NL. Comparative thresholds for acetylcholinesterase inhibition and behavioral impairment in coho salmon exposed to chlorpyrifos. Environ Technol Chem. 2005;24(1):136-45.

29. Tierney KB, Sampson JL, Ross PS, Sekela MA, Kennedy CJ. Salmon olfaction is impaired by an environmentally realistic pesticide mixture. Environ Sci Technol. 2008;42:4996-5001.

30. Anderson TD, Zhu KY. Synergistic and antagonistic effects of atrazine on the toxicity of organophosphorodithioate and organophosphorothioate insecticides to *Chrionomus tentans* (Diptera: Chironomidae). Pestic Biochem Physiol. 2004;80:54-64.

31. Laetz CA, Baldwin DH, Collier TK, Hebert V, Stark JD, Scholz NL. The synergistic toxicity of pesticide mixtures: implications for risk assessment and the conservation of endangered Pacific salmon. Environ Health Perspect. 2009 Mar;117(3)348-53.

32. Laetz CA, Baldwin DH, Hebert VR, Stark JD, Scholz NL. Interactive neurobehavioral toxicity of diazinon, malathion, and ethoprop to juvenile coho salmon. Environ Sci Techol. 2013;47:2925-31

33. National Marine Fisheries Service. Endangered Species Act Section 7 Consultation, Biological Opinion on the Environmental Protection Agency registration of pesticides containing chlorpyrifos, diazinon, and malathion. Final biological opinion. Silver Spring (MD): National Oceanic and Atmospheric Administration, National Marine Fisheries Service; 2008 Nov. http://www.nmfs.noaa.gov/pr/pdfs/pesticide_biop.pdf.

34. Sandahl JF, Baldwin DH, Jenkins JJ, Scholz NL. Odor-evoked field potentials as indicators of sublethal neurotoxicity in juvenile coho salmon (*Oncorhynchus kisutch*) exposed to copper, chlorpyrifos, or esfenvalerate. Can J Fish Aquat Sci. 2004;61:404-13.

35. Macneale KH, Spromberg JA, Baldwin DH, Scholz NL. A modeled comparison of direct and food web-mediated impacts of common pesticides on Pacific Salmon. PloS One. 2014;9(3):e92436.

36. Ferrando MD, Andreu-Moliner E. Acute lethal toxicity of some pesticides to *Brachionus calyciflorus* and *Brachionus plicatilis*. Bull Environ Contam Toxicol. 1991;47:479-84.

37. Schmitz A, Nagel R. Influence of 3,4-Dichloroaniline (3,4-DCA) on benthic invertebrates in indoor experimental streams: Ecotoxicol Environ Saf. 1995;30:63-71.

38. Risks of Iprodione use to federally threatened California red-legged frog [Internet]. U.S. Environmental Protection Agency; 2009 [cited 2015 Sep 11]. Available from: http://nepis.epa.gov/Exe/ZyPDF.cgi/P1007YS9.PDF?Dockey=P1007YS9.PDF

39. Kreutzweiser DP, Holmes SB, Eichenberg DC. Influence of exposure duration on the toxicity of triclopyr ester to fish and aquatic insects. Arch Environ Contam Toxicol. 1994;26:124-9.

40. Norris LA, Montgomery ML, Warren LE. Triclopyr persistence in western Oregon hill pastures. Bull Environ Contam Toxicol. 1987;39:134-141.

41. Thompson DG, Staznik B, Fontaine DD, Mackay T, Oliver GR, Troth J. Fate of triclopyr ester (Release®) in a boreal forest stream. Environ Toxicol Contam. 1991;10:619-32.

42. Triclopyr technical fact sheet [Internet]. Corvallis (OR): National Pesticide Information Center; c.2002 [updated 2002; cited 2015 Sep 30]. Available from: http://npic.orst.edu/factsheets/archive/triclotech.pdf

43. Boscalid pesticide fact sheet [Internet]. U.S. Environmental Protection Agency; 2003 [cited 2015 Sep 11]. Available from: http://www3.epa.gov/pesticides/chem_search/reg_actions/registration/fs_PC-128008_01-Jul-03.pdf

44. Aubee C, Lieu D. Environmental fate and ecological risk assessment for boscalid – new uses on alfalfa and citrus [Internet]. U.S. Environmental Protection Agency, Office of Pesticide Programs; 2010 Feb. [cited 2015 Nov 10]. Available from: http://archive.epa.gov/pesticides/chemicalsearch/chemical/foia/web/pdf/128008/128008-2010-02-19a.pdf

45. Mortensen AS, Arukwe A. The persistent DDT metabolite, 1,1-dichloro-2,2-bis(*p*-chlorophenyl)ethylene, alters thyroid hormone-dependent genes, hepatic cytochrome P4503A, and pregnane X receptor gene expressions in Atlantic salmon (*Salmo salar*) parr. Environ Toxicol Chem. 2006;25(6):1607-15. http://www.ncbi.nlm.nih.gov/pubmed/16764480

46. Eisler R. Chlordane hazards to fish, wildlife, and invertebrates: A synoptic review. Laurel (MD): U.S. Fish and Wildlife Service, Patuxent Wildlife Research Center; 1990 Jul. Biological Report 85(1.21).

47. Phipps GL, Mattson VR, Ankley GT. Relative sensitivity of three freshwater benthic macroinvertebrates to ten contaminants. Arch Environ Contam Toxicol. 1995;28:281-286.

48. Wang WX, Fisher NS. Assimilation efficiencies of chemical contaminants in aquatic invertebrates: A synthesis. Environ Toxicol Chem. 1999;18(9):2034-45.

49. Manchester-Neesvig JB, Valters K, Sonzogni WC. Comparison of PBDEs and PCBs in Lake Michigan salmonids. Environ Sci Technol. 2001;35:1072-7.

50. Arkoosh MR, Boylen D, Dietrich J, Anulacion BF, Ylitalo G, Bravo CF, et al. Disease susceptibility of salmon exposed to polybrominated diphenyl ethers (PBDEs). Aquat Toxicol. 2010;98:51-9.

51. Yu L, Lam JCW, Guo Y, Wu RSS, Lam PKS, Zhou B. Parental transfer of polybrominated diphenyl ethers (PBDEs) and thyroid endocrine disruption in zebrafish. Environ Sci Technol. 2011;45:10652-9.

52. Mariussen E, Fjeld E, Breivik K, Steinnes E, Borgen A, Kjellberg G, et al. Elevated levels of polybrominated diphenyl ethers (PBDEs) in fish from Lake Mjøsa, Norway. Sci Total Environ. 2008;390(1):132-41.

53. Chou CT, Hsiao YC, Ko, FC, Cheng JO, Cheng YM, Chen TH. Chronic exposure of 2,2',4,4'-tetrabromodiphenyl ether (PBDE-47) alters locomotion behavior in juvenile zebrafish (*Danio rerio*). Aquat Toxicol. 2010;98:388-95.

54. Dietrich JP, Strickland SA, Hutchinson GP, Van Gaest AL, Krupin AB, Ylitalo GM, et al. Assimilation efficiency of PBDE congeners in chinook salmon. Environ Sci Technol. 2015;49:3878-86.

55. King KA, Grue CE, Grassley JM, Fisk RJ. Pesticides in urban streams and early life stages of Pacific coho salmon. Environ Toxicol Chem. 2013;32(4):920-31.

56. King KA, Grue CE, Grassley JM, Fisk RJ Conquest LL. Growth and survival of Pacific coho salmon smolts exposed as juveniles to pesticides within urban streams in western Washington, USA. Environ Toxicol Chem. 2014;33(7)1596-606.

57. Maas-Hebner KG, Hughes RM, Schreck CB. Wild salmonids in the urban environment – lethal and sublethal effects. In: Yeakley JA, Maas-Hebner KG, Hughes, RM, editors. Wild salmonids in the urbanizing Pacific Northwest. Springer: New York; 2014. pp. 169-182.

58. Ross PS, Kennedy CJ, Shelley LK, Tierney KB, Patterson DA, Fairchild WI, et al. The trouble with salmon – relating pollutant exposure to toxic effect in species with transformational life histories and lengthy migrations. Can J Fish Aquat Sci. 2013;70:1252-64.

59. Baldwin DH, Spromberg JA, Collier TK, Scholz NL. A fish of many scales: extrapolating sublethal pesticide exposures to the productivity of wild salmon populations. Ecol Appl. 2009;18(8):2004-15.

60. Spromberg JA, Meador JP. Relating results of chronic toxicity responses to population-level effects – modeling effects on wild Chinook salmon populations: Integr Environ Assess Manag. 2005;1(1):9-21.

61. Cuffney TF, Wallace JB, Webster JR. Pesticide manipulation of a headwater stream: invertebrate responses and their significance for ecosystem processes. J Freshw Invert Biol. 1984;3:153-71.
