# Supplementary material for: Year-Round Monitoring of Contaminants in Neal and Rogers Creeks, Hood River Basin, Oregon, 2011-12, and Assessment of Risks to Salmonids
Source: PLoS One. 2016 Jun 27;11(6):e0158175. doi: 10.1371/journal.pone.0158175 (PMC4922572; doi:10.1371/journal.pone.0158175)
Supplement: S2 Text — (PDF) [file pone.0158175.s007.pdf]

# S2 Text. Discussion of comparisons between passive and discrete sample data

Concentration data from passive samplers are time-weighted averages (TWAs); values represent the average concentration at any time in the deployment period. Therefore, they are not strictly comparable to point-in-time concentrations measured from discrete samples. Adding to this difficulty are differences in the nature of the dissolved chemical (freely dissolved for passive samplers versus total dissolved for a filtered discrete sample) and potential methodological issues related to filtering discrete samples including absorption of chemicals to the filtration equipment and efficiencies in filtration [1].

Ellis et al. [2] found SPMD-derived water concentrations of 4,4'-DDD, 4,4'-DDE, and 4,4'-DDT were 1.1 to 2.3 times lower than direct measurements of samples collected using large-volume solid-phase extraction. Jacquet et al. [3] found SPMD-derived water concentrations of PCBs to be 0.8 to 2.1 (average 1.5) times greater than the nominal water concentrations in laboratory studies. The degree of variance changed depending on the calculation method used; however, even in the worst case, the agreement was considered quite acceptable. Limited comparisons have been done with POCIS results. Atrazine and diuron estimates from POCIS were found to agree within a factor of 3 with discrete measurements [4,5].

Results from passive sampling devices have been occasionally considered semi-quantitative by some groups as the TWA water concentrations are not a direct measurement, but rather an estimate based off either experimentally or theoretically-derived sampling rates, octanol-water partition coefficients, $K_{ow}$, and membrane-water partition coefficients, $K_{sw}$ [6]. Similarly, the representativeness of a discrete sample's results of single point in space and time is considered semi-quantitative with respect to the average concentration of a chemical in the water body.

The uncertainty in estimates from passive samplers exists not in the analysis of chemical residues in the sampler, but in the variability of $K_{sw}$ measurements [1]. Field conditions such as water flow at the SPMD surface, water temperature, and the build-up of a biofilm on the sampler's surface all affect the sampling kinetics into a passive sampler. The use of performance reference compounds (PRCs) has greatly increased the reliability and accuracy of SPMD-derived water concentration estimates by providing a means of accounting for these site-specific environmental variables that can affect rates of chemical partitioning into the sampler [6]. Unfortunately, the PRC approach has not been successfully adapted for use with the POCIS, except in a few specific applications [7]. Until the sampling kinetics of the POCIS are better understood, data generated from it will be less quantitative than other passive sampling devices, but will be highly useful when comparable data from discrete samples is logistically and/or technologically impossible to obtain.

# References


1. Booij K, Robinson CD, Burgess RM, Mayer P, Roberts CA, Ahrens L, et al. Passive sampling in regulatory chemical monitoring of nonpolar organic compounds in the aquatic environment. Environ Sci Technol. 2016:50:3-17.
2. Ellis SG, Booij K, Kaputa M. Comparison of semipermeable membrane device (SPMD) and large-volume solid-phase extraction techniques to measure water concentrations of 4,4'-DDT, 4,4'-DDE, and 4,4'-DDD in Lake Chelan, Washington. Chemosphere. 2008:72:1112-1117.
3. Jacquet R, Miège C, Smedes F, Tixier C, Tronczynski J, Togola, et al. Comparison of five integrative samplers in laboratory for the monitoring of indicator and dioxin-like polychlorinated biphenyls in water. Chemosphere. 2014:98:18-27.
4. Alvarez DA, Petty JD, Huckins JN, Jones-Lepp TL, Getting DT, Goddard JP, Manahan SE. Development of a passive, in situ, integrative sampler for hydrophilic organic contaminants in aquatic environments. Environ Toxicol Chem. 2004:23:1640-1648.
5. Petty JD, Huckins JN, Alvarez DA, Brumbaugh WG, Cranor WL, Gale RW, et al. A holistic passive integrative sampling approach for assessing the presence and potential impacts of waterborne environmental contaminants. Chemosphere. 2004:54:695-705.
6. Huckins JN, Petty JD, Booij K. Monitors of organic chemicals in the environment: semipermeable membrane devices. Springer, New York, 2006.
7. Harman C, Allan IJ, Vermeirssen ELM. Calibration and use of the polar organic chemical integrative sampler-A critical review. Environ Toxicol Chem. 2012:31:2724-2738.
